# Data mining and safety analysis of dual orexin receptor antagonists (DORAs): a real-world pharmacovigilance study based on the FAERS database

**DOI:** 10.3389/fphar.2024.1436405

**Published:** 2024-08-06

**Authors:** Manxue Jiang, Hao Li, Lingti Kong

**Affiliations:** ^1^ Department of Pharmacy, The First Affiliated Hospital of Bengbu Medical University, Bengbu, China; ^2^ School of Pharmacy, Bengbu Medical University, Bengbu, China; ^3^ Institute of Emergency and Critical Care Medicine, The First Affifiliated Hospital of Bengbu Medical University, Bengbu, China

**Keywords:** dual orexin receptor antagonists (DORAs), insomnia, FDA adverse event reporting system, adverse drug events, data mining

## Abstract

**Objective:**

Using the Food and Drug Administration Adverse Event Reporting System (FAERS) database, four signal detection methods were applied to mine adverse drug events (ADEs) related to use of dual orexin receptor antagonists (DORAs) to provide reference for safe clinical use.

**Research design and Methods:**

Data collected from Q3rd 2014 to Q4th 2023 were obtained from the FAERS database. According to the preferred terminology (PT) and systematic organ classification (SOC) of MedDRA v.26.0, the reporting odds ratio (ROR), proportional reporting ratio (PRR), multi-item gamma Poisson shrinker (MGPS), and Bayesian confidence propagation neural network (BCPNN) were used to detect ADE signals.

**Results:**

A total of 11,857 DORAs-related adverse reactions were detected, reported with suvorexant, lemborexant, and daridorexant as the main suspected drugs was 8717584, and 2556, respectively. A higher proportion of females than males were reported (57.27% vs. 33.04%). The top 20 positive PT signals from three DORAs showed that “sleep paralysis” ranked first. “Brain fog” was stronger following daridorexant but was not detected for the other two drugs, and “sleep sex” and “dyssomnia” were stronger in suvorexant but not in the other two drugs. Additionally, some PTs occurred that were not included in drug instructions, such as “hangover” and “hypnagogic hallucination.”

**Conclusion:**

In this study, four algorithms (ROR, PRR, BCPNN, and MGPS) were used to mine the safety signals of DORAs. We identified some potential ADE signals that can promote the rational use of DORAs and improve their safety.

## 1 Introduction

Insomnia is one of the most common diseases in the population and can have a negative impact on the lives and work of patients. Approximately 30% of adults worldwide experience varying degrees of insomnia (Brownlow et al., 2020; Robinson et al., 2022). Symptoms of insomnia include difficulty falling asleep, poor sleep quality, and early awakening, which can cause patients to feel fatigue, somnolence, and difficulty concentrating during the day. Long-term insomnia may also cause psychological problems such as anxiety and depression, and may even affect physical health, increasing the risk of cardiovascular disease, diabetes, and other diseases (Kwok et al., 2018; Chasens et al., 2021; Boer et al., 2023). Therefore, it is very important for individuals experiencing insomnia to receive effective treatment measures in a timely manner. This includes improving the sleep environment, adjusting daily routines, using non-pharmacological treatments such as relaxation techniques, and using medications to alleviate symptoms. The first-line treatment for insomnia is cognitive behavioral therapy for insomnia (CBT-I), which does not require medication intervention and has fewer side effects. However, it requires guidance from professional psychotherapists, has a long treatment cycle, and requires active cooperation from patients, which limits its widespread clinical application (Schutte-Rodin et al., 2008; Thomas et al., 2016; Riemann et al., 2023). When patients experience symptoms such as difficulty falling asleep, frequent and persistent awakenings at night, and early awakenings that persist for several times a week over a period of 3 months, and self-regulation fails, pharmacological treatment is often used to treat patients who have not responded to non pharmacological treatment (Sateia, 2014; Riemann et al., 2023).

Dual orexin receptor antagonists (DORAs) are a newly introduced medication for the treatment of insomnia. Compared with other insomnia drugs, the biggest difference of DORAs lies in their mechanism of action. Traditional insomnia drugs, such as benzodiazepines (such as estazolam and nonbenzodiazepine drugs (such as zaleplon), mainly induce sedative and hypnotic effects by enhancing the effect of GABA neurotransmitters (Madari et al., 2021; Grassi et al., 2023; Rosenberg et al., 2023). DORAs, in contrast, reduce arousal by blocking orexin receptors (Rocha et al., 2023). The advantage of DORAs is that they are less prone to dependency and tolerance, and have relatively fewer side effects (Rosenberg et al., 2019; Sarathi Chakraborty et al., 2023).

Currently, the three drugs on the market are suvorexant, which was introduced in August 2014, lemborexant, which was launched in December 2019, and daridorexant, which was approved for marketing in January 2022 (Yang, 2014; Scott, 2020; Markham, 2022). In a systematic review and network meta-analysis (NMA) investigating the efficacy and safety of different doses of FDA-approved DORAs for the treatment of primary insomnia, it was found that suvorexant 20 mg, lemborexant 5 mg, lemborexant 10 mg, and daridorexant 50 mg represent suitable approaches for insomnia (Xue et al., 2023). A study on suvorexant also found that participants fell asleep less and woke up less during the night, and also noted that a lower likelihood of addiction compared to other categories of sleep drugs was a benefit (Bennett et al., 2014). In a 40-week extension study, daridorexant improved sleep and daytime functioning while maintaining a favorable safety profile and well tolerated in patients with insomnia disorder (Kunz et al., 2023). And in a 12-month global, multicenter, randomized, double-blind, parallel group Phase 3 study, lemborexant provided significant benefits on sleep onset and sleep maintenance in individuals with insomnia disorder *versus* placebo, and was well tolerated (Kärppä et al., 2020).

However, each drug has its potential adverse drug events (ADEs), which are an important topic in drug safety research and an unavoidable challenge in medical practice. Although clinical trials are conducted before any drug is marketed, due to the limitation of sample size, it is not possible to comprehensively predict the potential adverse reactions of the drug in a large population. Therefore, post-marketing studies of drugs require extensive adverse reaction monitoring and analysis to ensure the safety of the drug. FAERS is a system that collects and analyzes adverse event reports submitted by healthcare professionals, consumers, and manufacturers. The system is also publicly available through the FDA website, which increases transparency and allows researchers and the public to access information for further analysis (Hu et al., 2024; Liu et al., 2024). The purpose of this study was to use the Food and Drug Administration Adverse Event Reporting System (FAERS) database to conduct signal mining on DORAs-related ADEs, to provide reference for clinical safe medication.

## 2 Methods

### 2.1 Data sources

**FIGURE 1:**
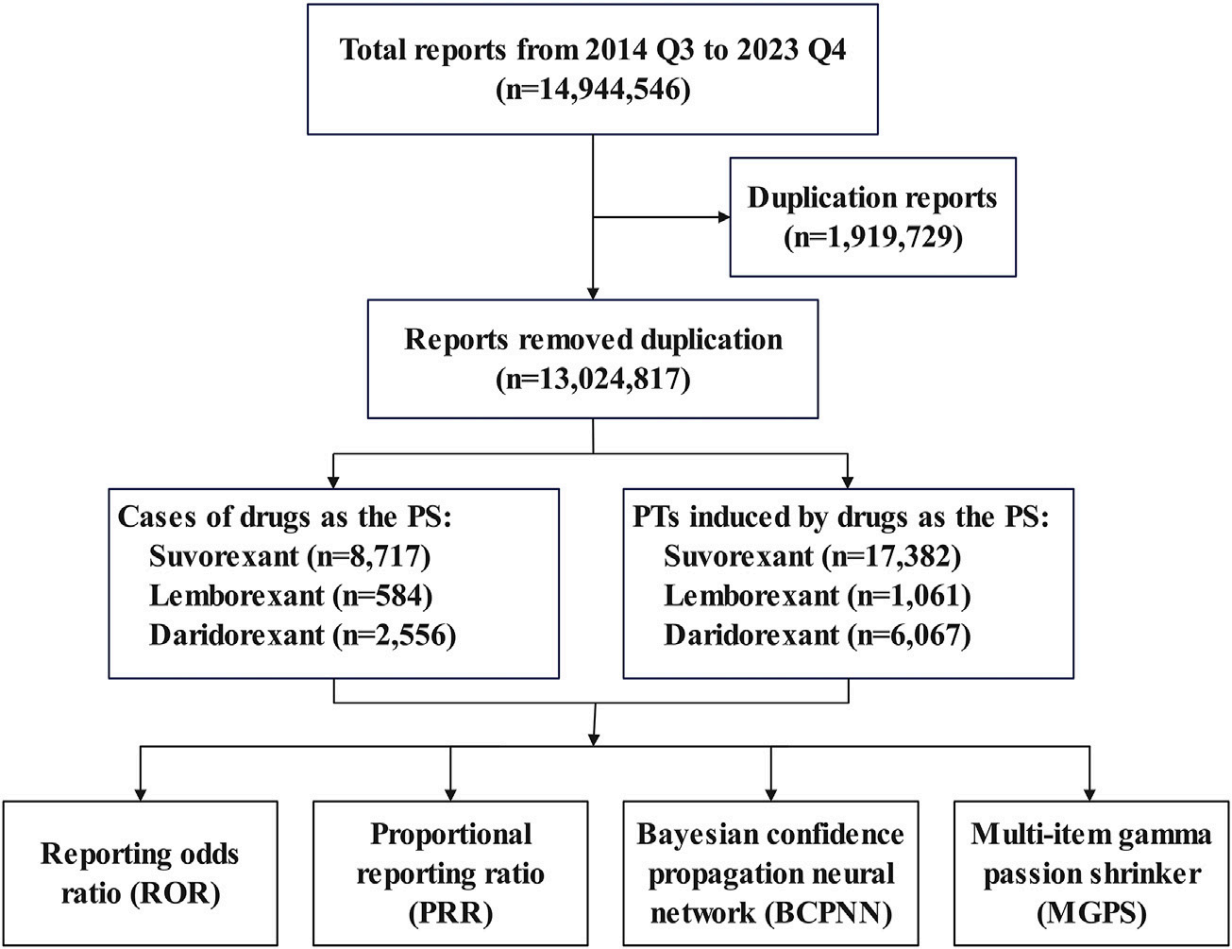
Data filtering flowchart.

The data for this study were sourced from ASCII files in the FAERS database (provided quarterly) (Zhai et al., 2019; Vestergaard Kvist et al., 2021), selecting data from Q3rd 2014 to Q4th 2023. The dataset consisted of seven data tables: patient demographic and administrative information (DEMO), drug information (DRUG), adverse events (REAC), patient outcomes (OUTC), report sources (RPSR), drug therapy start and end dates (THE), and indications for use or diagnosis (INDI). We removed duplicate data based on the case ID and primary ID. We removed duplicates from the data according to the following criteria: 1) If the case IDs were the same, then the report with the larger primary ID was selected; 2) if the primary ID was the same, then this was considered to be an error and these records were excluded. The data processing flow is shown in Figure 1.

### 2.2 Data filtering

The search in the FAERS database were performed using the generic names (“suvorexant,” “lemborexant,” and “daridorexant”) and the product names (“Belsomra,” “Dayvigo,” and “Quviviq”) as keywords. Only the role_cod was PS (Primary suspected) were included in this study. ADEs were described and classified using the preferred terminology (PT) and the system organ category (SOC) in the Medical Dictionary for Regulatory Activities (MedDRA^®^ v.26.0) terminology set (Tieu and Breder, 2018; Zhou et al., 2021). MedDRA^®^ is the international medical terminology developed under the auspices of the International Council for Harmonisation of Technical Requirements for Pharmaceuticals for Human Use (ICH) to classify adverse event information associated with the use of biopharmaceuticals and other medical products (e.g., medical devices and vaccines). The MedDRA^®^ trademark is registered by the ICH.

### 2.3 Statistical analysis

To evaluate whether the drug was significantly correlated with ADEs, we calculated the reporting odds ratio (ROR), proportional reporting ratio (PRR), information component (IC), and empirical Bayes geometric mean (EBGM) based on disproportionality analysis and Bayesian analysis. The ROR and PRR methods are relatively simple to calculate and allow for an estimate of relative risk. Among them, the PRR method can better control the impact of reporting biases and comorbid factors (Zhai et al., 2021). In order to avoid the high sensitivity of these two methods, this study combined signal detection methods such as PRR, ROR, IC, and EBGM to screen overlapping signals, which can reduce the number of false positive and false negative signals with good sensitivity (Jaffa et al., 2015; Wang et al., 2021). The calculations used by the four data mining algorithms were shown in Table 1 (Hauben and Zhou, 2003; Böhm et al., 2021; Shao et al., 2022; Zhao et al., 2023).

**TABLE 1:** Summary of the main algorithms used for signal detection.

Algorithm	Equation	Positive criteria
ROR	$ROR=\frac{a}{c}\times \frac{b}{d}$ $95\%CI=e^{\ln\;\left(ROR\right)\pm 1.96\sqrt{\frac{1}{a}+\frac{1}{b}+\frac{1}{c}+\frac{1}{d}}}$	lower limit of 95%CI>1 a≥3
PRR	$PRR=\frac{a}{\left(a+b\right)}÷\frac{c}{\left(c+d\right)}$ $\chi^{2}=\frac{{\left(ad-bc\right)}^{2}\times \left(a+b+c+d\right)}{\left(a+b\right)\left(c+d\right)\left(a+c\right)\left(d+b\right)}$ $95\%CI=e^{\ln\left(PRR\right)\pm 1.96\sqrt{\frac{1}{a}-\frac{1}{a+b}+\frac{1}{c}-\frac{1}{c+d}}}$	PRR≥2 $\chi^{2}\geq 4,a\geq 3$
BCPNN	$V\left(IC\right)=\frac{1}{{\left(\ln\right)}^{2}}\left[\frac{b+c+d+\gamma -1}{\left(a+1\right)(1+a+b+c+d+\gamma }+\frac{c+d+1}{\left(a+b+1\right)\left(a+b+c+d+3\right)}+\frac{b+d+1}{\left(a+c+1\right)\left(a+b+c+d+3\right)}\right]$ $\gamma =\frac{{\left(a+b+c+d+2\right)}^{2}}{\left(a+b+1\right)\left(a+c+1\right)}$ $E\left(IC\right)=\log_{2}\frac{\left(a+1\right){\left(a+b+c+d+2\right)}^{2}}{\left(a+b+c+d+\gamma \right)\left(a+b+1\right)\left(a+c+1\right)}$ $IC025=E\left(IC\right)-2\sqrt{V\left(IC\right)}$ $95\%CI=e^{\ln\;\left(IC\right)\pm 1.96\sqrt{\frac{1}{a}+\frac{1}{b}+\frac{1}{c}+\frac{1}{d}}}$	IC025>0
MGPS	$EBGM=\frac{a\left(a+b+c+d\right)}{\left(a+c\right)\left(a+b\right)}$ $EBGM05=e^{\ln\left(EBGM\right)-1.96\sqrt{\frac{1}{a}+\frac{1}{b}+\frac{1}{c}+\frac{1}{d}}}$ $95\%CI=e^{\ln\;\left(EBGM\right)\pm 1.96\sqrt{\frac{1}{a}+\frac{1}{b}+\frac{1}{c}+\frac{1}{d}}}$	EBGM05≥2 N>0

Abbreviations: ROR, reporting odds ratio; PRR, proportional reporting ratio; BCPNN, bayesian confidence propagation neural network; MGPS, multi-item gamma passion shrinker; IC, information component; EBGM, empirical Bayes geometric mean; a, number of reports arising from the suspect adverse events (AE) and the suspect drug; b, number of reports arising from the suspect AE, and all other drugs; c, number of reports arising from the suspect drug and other ADEs; d, number of reports arising from other drugs and other ADEs; CI, confidence interval; χ^2^

Chi-squared; IC025, lower limit of 95% two-sided CI, of the IC; EBGM05, lower limit of 95% one-sided CI, of EBGM.

Descriptive and difference statistics were analyzed using SPSS (v.26.0) and the data were visualized using R (version 4.3.1).

## 3 Results

### 3.1 Descriptive analysis

As shown in Table 2, there were a total of 11,857 reports of ADEs related to DORAs, including 8,717 cases involving suvorexant (73.52%), 584 cases involving lemborexant (4.93%), and 2,556 cases involving daridorexant (21.56%). Among patients of known age, those aged 18 to <65 years had the highest proportion, followed by patients aged 65–85 years. Among patients of known sex, females had significantly higher rate than males for all three DORAs. Most ADEs were reported by consumers, with the reporting countries coming primarily from the United States, followed by Japan.

**TABLE 2:** Clinical characteristics of the patients.

Characteristic	Suvorexant	Lemborexant	Daridorexant	Overall
	(N = 8,717)	(N = 584)	(N = 2,556)	(N = 11,857)
Sex				
Female	4,783 (54.87%)	326 (55.82%)	1,681 (65.77%)	6790 (57.27%)
Male	2,913 (33.42%)	202 (34.59%)	803 (31.42%)	3918 (33.04%)
Missing	1,021 (11.71%)	56 (9.59%)	72 (2.82%)	1,149 (9.69%)
Age (years)				
<18	26 (0.30%)	3 (0.51%)	0 (0.00%)	29 (0.24%)
18–64.9	1819 (20.87%)	185 (31.68%)	406 (15.88%)	2,410 (20.33%)
6585	1,581 (18.14%)	124 (21.23%)	289 (11.31%)	1994 (16.82%)
>85	269 (3.09%)	17 (2.91%)	14 (0.55%)	300 (2.53%)
Missing	5,022 (57.61%)	255 (43.66%)	1847 (72.26%)	7,124 (60.08%)
Reporter type				
Consumer	6435 (73.82%)	193 (33.05%)	2,287 (89.48%)	8,915 (75.19%)
Health-professional	2,198 (25.22%)	388 (66.44%)	266 (10.41%)	2,852 (24.05%)
Other/Missing	84 (0.96%)	3 (0.51%)	3 (0.12%)	90 (0.76%)
Reporter country				
America	7,923 (90.89%)	418 (71.58%)	2,549 (99.73%)	10,890 (91.84%)
Japan	753 (8.64%)	139 (23.80%)	0 (0.00%)	892 (7.52%)
Canada	1 (0.01%)	18 (3.08%)	0 (0.00%)	19 (0.16%)
Australia	25 (0.29%)	5 (0.86%)	0 (0.00%)	30 (0.25%)
Other/Missing	15 (0.17%)	4 (0.68%)	7 (0.27%)	26 (0.22%)

### 3.2 Number of positive signals

**FIGURE 2:**
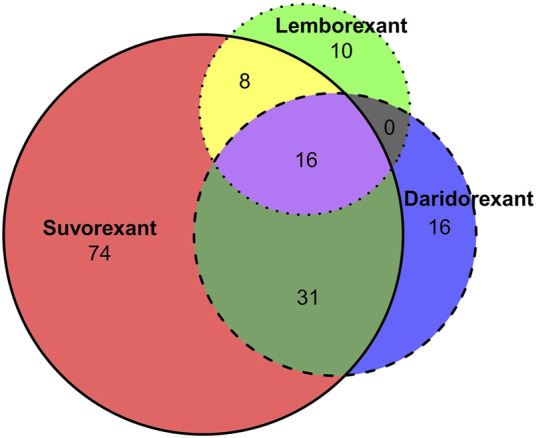
Intersection of the number of PT signals.

In this study, ADE was considered a positive signal when four indicators were met simultaneously. According to the signal calculation method in Table 1, the number of positive signals detected for suvorexant, lemborexant, and daridorexant were 129, 34, and 63, respectively. The intersection was shown in Figure 2, where 16 signals are present in all three drugs.

### 3.3 SOCs involved in positive signals

Regrouping of positive signals using MedDRA for SOCs is shown in Table 3. The number of SOCs involved for suvorexant, lemborexant, and daridorexant were 14, 7, and 11, respectively. “General disorders and administration site conditions,” “psychiatric disorders,” and “nervous system disorders” were the top three in terms of frequency of the three drugs.

**TABLE 3:** Systematic classification of organs involved in positive signals.

SOCs	Suvorexant	Lemborexant	Daridorexant	Overall
General disorders and administration site conditions	4,123 (42.07%)	120 (24.14%)	1,182 (30.59%)	5,425 (38.31%)
Psychiatric disorders	3663 (37.38%)	172 (34.61%)	1,419 (36.72%)	5,254 (37.10%)
Nervous system disorders	1,287 (13.13%)	174 (35.01%)	399 (10.33%)	1860 (13.13%)
Injury, poisoning and procedural complications	258 (2.63%)	18 (3.62%)	361 (9.34%)	637 (4.50%)
Product issues	156 (1.59%)	0 (0.00%)	254 (6.57%)	410 (2.90%)
Cardiac disorders	106 (1.08%)	0 (0.00%)	48 (1.24%)	154 (1.09)
Gastrointestinal disorders	73 (0.74%)	0 (0.00%)	42 (1.09%)	115 (0.81%)
Social circumstances	43 (0.44%)	0 (0.00%)	50 (1.29%)	93 (0.66%)
Immune system disorders	0 (0.00%)	0 (0.00%)	72 (1.86%)	72 (0.51%)
Skin and subcutaneous tissue disorders	31 (0.32%)	0 (0.00%)	20 (0.52%)	51 (0.36%)
Renal and urinary disorders	12 (0.12%)	0 (0.00%)	17 (0.44%)	29 (0.20%)
Respiratory, thoracic, and mediastinal disorders	21 (0.21%)	0 (0.00%)	0 (0.00%)	21 (0.15)
Infections and infestations	14 (0.14%)	5 (1.01%)	0 (0.00%)	19 (0.13%)
Investigations	9 (0.09%)	0 (0.00%)	0 (0.00%)	9 (0.06%)
Hepatobiliary disorders	0 (0.00%)	5 (1.01%)	0 (0.00%)	5 (0.04%)
Reproductive system and breast disorders	4 (0.04%)	0 (0.00%)	0 (0.00%)	4 (0.03)
Musculoskeletal and connective tissue disorders	0 (0.00%)	3 (0.60%)	0 (0.00%)	3 (0.02%)
Total	9800 (100.00%)	497 (100.00%)	3864 (100.00%)	14,161 (100.00)

### 3.4 Top 20 positive PT signals

The top 20 positive PTs in terms of signal intensity for suvorexant, daridorexant, and lemborexant are shown in Tables 4–6, respectively. A summary of the three DORAs is shown in Table 7. The greater the signal intensity, the stronger the correlation between the PT and the drug. For all three DORAs, the PT with the highest signal intensity was “sleep paralysis,” with prevalence rates of 2.60% (227/8,717), 10.96% (64/584), and 2.19% (56/2,556), respectively.

**TABLE 4:** Top 20 PTs in terms of signal intensity of suvorexant.

PTs	Frequency	ROR (95% CI)	PRR (χ^2^)	EBGM (EBGM05)	IC (IC025)
Sleep paralysis	227	620.18 (534.93–719.01)	612.09 (108,294.76)	478.84 (423.11)	8.90 (7.23)
Sleep sex	3	193.70 (59.49–630.73)	193.67 (528.40)	178.05 (66.30)	7.48 (5.75)
Labelled drug-food interaction issue	5	138.96 (56.27–343.14)	138.92 (643.87)	130.71 (61.35)	7.03 (5.34)
Abnormal sleep-related event	34	123.59 (87.46–174.64)	123.35 (3906.69)	116.84 (87.49)	6.87 (5.20)
Abnormal dreams	582	110.12 (101.2–119.84)	106.47 (58,013.19)	101.59 (94.65)	6.67 (5.00)
Hypnagogic hallucination	7	85.87 (40.35–182.75)	85.83 (564.83)	82.64 (43.93)	6.37 (4.69)
Nightmare	705	84.95 (78.68–91.72)	81.55 (54,106.87)	78.66 (73.77)	6.30 (4.63)
Somnambulism	131	81.69 (68.58–97.32)	81.09 (9993.72)	78.23 (67.58)	6.29 (4.62)
Dyssomnia	4	80.57 (29.70–218.55)	80.55 (303.11)	77.73 (33.73)	6.28 (4.59)
Product blister packaging issue	26	76.82 (51.94–113.61)	76.70 (1877.07)	74.15 (53.44)	6.21 (4.54)
Cataplexy	33	66.15 (46.77–93.55)	66.03 (2051.74)	64.13 (47.98)	6.00 (4.33)
Hangover	62	62.30 (48.38–80.22)	62.08 (3623.81)	60.40 (48.88)	5.92 (4.25)
Sleep-related eating disorder	14	52.75 (31.04–89.64)	52.71 (693.53)	51.50 (33.04)	5.69 (4.02)
Therapeutic response increased	13	50.10 (28.90–86.83)	50.06 (611.10)	48.97 (30.91)	5.61 (3.94)
Irregular sleep phase	3	49.89 (15.89–156.70)	49.88 (140.52)	48.80 (18.73)	5.61 (3.92)
Sleep terror	48	41.64 (31.28–55.42)	41.53 (1863.35)	40.77 (32.10)	5.35 (3.68)
Sleep attacks	4	40.47 (15.05–108.81)	40.46 (151.15)	39.74 (17.37)	5.31 (3.64)
Terminal insomnia	16	40.22 (24.52–65.96)	40.18 (600.32)	39.48 (26.10)	5.30 (3.63)
Therapeutic product effect prolonged	10	39.79 (21.28–74.37)	39.76 (371.16)	39.07 (23.15)	5.29 (3.62)
Middle insomnia	160	36.52 (31.22–42.73)	36.20 (5,388.50)	35.63 (31.24)	5.15 (3.49)

**TABLE 5:** Top 20 PTs in terms of signal intensity of lemborexant.

PTs	Frequency	ROR (95% CI)	PRR (χ^2^)	EBGM (EBGM05)	IC (IC025)
Sleep paralysis	64	2,507.72 (1932.8–3253.66)	2,356.52 (141,428.63)	2,211.70 (1778.68)	11.11 (9.44)
Cataplexy	4	128.27 (47.97–343.01)	127.79 (501.44)	127.34 (55.92)	6.99 (5.32)
Sleep terror	9	126.75 (65.69–244.57)	125.68 (1,109.43)	125.25 (72.27)	6.97 (5.30)
Nightmare	53	102.19 (77.5–134.75)	97.14 (5,031.82)	96.88 (76.86)	6.60 (4.93)
Abnormal dreams	32	94.56 (66.49–134.49)	91.74 (2,865.68)	91.51 (68.15)	6.52 (4.85)
Somnambulism	7	69.06 (32.82–145.33)	68.61 (465.56)	68.49 (36.75)	6.10 (4.43)
Hangover	3	48.08 (15.47–149.42)	47.94 (137.72)	47.88 (18.54)	5.58 (3.91)
Middle insomnia	12	44.32 (25.08–78.31)	43.83 (501.70)	43.77 (27.18)	5.45 (3.78)
Altered state of consciousness	13	37.42 (21.65–64.67)	36.97 (454.63)	36.93 (23.36)	5.21 (3.54)
Initial insomnia	4	27.68 (10.36–73.91)	27.58 (102.39)	27.56 (12.11)	4.78 (3.12)
Paralysis	5	22.46 (9.32–54.08)	22.36 (101.96)	22.34 (10.71)	4.48 (2.81)
Hallucination, auditory	5	20.77 (8.62–50.01)	20.67 (93.57)	20.66 (9.90)	4.37 (2.70)
Neuroleptic malignant syndrome	3	17.51 (5.64–54.38)	17.46 (46.54)	17.45 (6.76)	4.13 (2.46)
Somnolence	52	16.29 (12.33–21.53)	15.55 (709.63)	15.54 (12.31)	3.96 (2.29)
Restless legs syndrome	4	13.36 (5.00–35.66)	13.31 (45.53)	13.30 (5.85)	3.73 (2.07)
Intentional overdose	14	13.20 (7.79–22.36)	13.04 (155.68)	13.03 (8.38)	3.70 (2.04)
Pneumonia aspiration	5	12.24 (5.08–29.48)	12.19 (51.36)	12.18 (5.84)	3.61 (1.94)
Restlessness	7	12.17 (5.79–25.59)	12.10 (71.27)	12.09 (6.49)	3.60 (1.93)
Poor quality sleep	4	10.66 (3.99–28.46)	10.63 (34.88)	10.62 (4.67)	3.41 (1.74)
Hallucination	12	10.19 (5.77–18.00)	10.08 (98.28)	10.08 (6.26)	3.33 (1.67)

**TABLE 6:** Top 20 PTs in terms of signal intensity of daridorexant.

PTs	Frequency	ROR (95% CI)	PRR (χ^2^)	EBGM (EBGM05)	IC (IC025)
Sleep paralysis	56	360.94 (275.41–473.03)	357.62 (18,844.05)	338.44 (269.90)	8.40 (6.73)
Labelled drug-food interaction issue	3	233.09 (73.61–738.09)	232.97 (668.19)	224.69 (85.65)	7.81 (6.12)
Hypervigilance	18	210.30 (131.40–336.57)	209.68 (3617.71)	202.94 (136.92)	7.66 (5.99)
Hangover	64	185.77 (144.70–238.5)	183.83 (11,307.41)	178.63 (144.94)	7.48 (5.81)
Hypnagogic hallucination	5	173.91 (71.49–423.03)	173.76 (835.77)	169.12 (80.38)	7.40 (5.72)
Nightmare	387	134.70 (121.40–149.45)	126.17 (47,136.40)	123.71 (113.41)	6.95 (5.28)
Product availability issue	234	109.32 (95.82–124.71)	105.14 (23,748.44)	103.43 (92.63)	6.69 (5.03)
Sleep-related eating disorder	9	96.42 (49.90–186.33)	96.28 (835.83)	94.84 (54.65)	6.57 (4.90)
Abnormal dreams	175	91.34 (78.51–106.27)	88.74 (14,974.91)	87.52 (77.11)	6.45 (4.79)
Somnambulism	50	87.43 (66.06–115.70)	86.71 (4,179.10)	85.55 (67.67)	6.42 (4.75)
Parasomnia	6	85.09 (38.01–190.49)	85.00 (491.46)	83.88 (42.74)	6.39 (4.72)
Brain fog	44	80.13 (59.46–107.99)	79.56 (3370.66)	78.57 (61.21)	6.30 (4.63)
Abnormal sleep-related event	8	79.85 (39.74–160.45)	79.75 (614.32)	78.76 (43.93)	6.30 (4.63)
Sleep talking	11	78.77 (43.44–142.84)	78.63 (832.66)	77.67 (47.20)	6.28 (4.61)
Sleep terror	29	71.71 (49.68–103.49)	71.37 (1989.65)	70.58 (51.92)	6.14 (4.47)
Product packaging difficult to open	16	66.87 (40.83–109.51)	66.70 (1,024.55)	66.01 (43.69)	6.04 (4.38)
Autoscopy	4	65.23 (24.35–174.73)	65.18 (250.21)	64.53 (28.29)	6.01 (4.34)
Accident at home	3	61.10 (19.59–190.54)	61.07 (175.56)	60.49 (23.36)	5.92 (4.24)
Middle insomnia	81	52.78 (42.35–65.78)	52.09 (4,026.71)	51.67 (42.98)	5.69 (4.02)
Therapeutic product effect variable	26	42.20 (28.67–62.11)	42.02 (1,034.37)	41.75 (30.21)	5.38 (3.72)

**TABLE 7:** Top 20 PTs of the ROR signal intensities of DORAs.

PTs	Frequency	ROR (95% CI)	PRR (χ^2^)	EBGM (EBGM05)	IC (IC025)
Sleep paralysis	347	789.30 (693.44–898.42)	778.14 (179,553.23)	519.10 (465.80)	9.02 (7.35)
Labelled drug-food interaction issue	8	163.87 (79.08–339.58)	163.82 (1,171.31)	148.31 (80.61)	7.21 (5.52)
Sleep sex	3	137.34 (42.18–447.19)	137.32 (373.06)	126.27 (47.02)	6.98 (5.25)
Hypnagogic hallucination	13	117.01 (66.58–205.62)	116.95 (1,389.97)	108.84 (67.91)	6.77 (5.09)
Abnormal sleep-related event	43	112.48 (82.52–153.32)	112.28 (4,423.47)	104.79 (80.87)	6.71 (5.04)
Abnormal dreams	789	107.57 (99.98–115.74)	104.14 (75,565.88)	97.67 (91.87)	6.61 (4.94)
Nightmare	1,145	100.80 (94.83–107.14)	96.13 (101,577.02)	90.60 (86.09)	6.50 (4.84)
Hangover	129	94.97 (79.47–113.49)	94.48 (11,248.60)	89.13 (76.78)	6.48 (4.81)
Somnambulism	188	84.50 (72.93–97.91)	83.86 (14,606.09)	79.62 (70.39)	6.32 (4.65)
Hypervigilance	26	76.14 (51.35–112.89)	76.06 (1836.17)	72.56 (52.19)	6.18 (4.51)
Sleep-related eating disorder	25	68.09 (45.61–101.64)	68.02 (1,581.75)	65.21 (46.64)	6.03 (4.36)
Hypnopompic hallucination	3	64.85 (20.43–205.85)	64.85 (181.04)	62.29 (23.70)	5.96 (4.26)
Dyssomnia	4	57.12 (21.06–154.94)	57.11 (212.71)	55.13 (23.92)	5.78 (4.10)
Product blister packaging issue	26	54.44 (36.82–80.51)	54.39 (1,316.54)	52.58 (37.90)	5.72 (4.05)
Cataplexy	38	54.24 (39.24–74.97)	54.16 (1916.03)	52.37 (39.94)	5.71 (4.04)
Sleep terror	86	53.74 (43.33–66.66)	53.56 (4,288.30)	51.81 (43.27)	5.70 (4.03)
Phobia of driving	3	49.15 (15.57–155.14)	49.15 (137.17)	47.67 (18.22)	5.58 (3.88)
Middle insomnia	253	41.39 (36.51–46.93)	40.98 (9616.52)	39.95 (35.97)	5.32 (3.65)
Sleep talking	23	41.28 (27.28–62.46)	41.24 (879.74)	40.20 (28.42)	5.33 (3.66)
Autoscopy	10	40.97 (21.86–76.78)	40.95 (379.80)	39.93 (23.61)	5.32 (3.65)

### 3.5 Difference between positive PT signals

**FIGURE 3:**
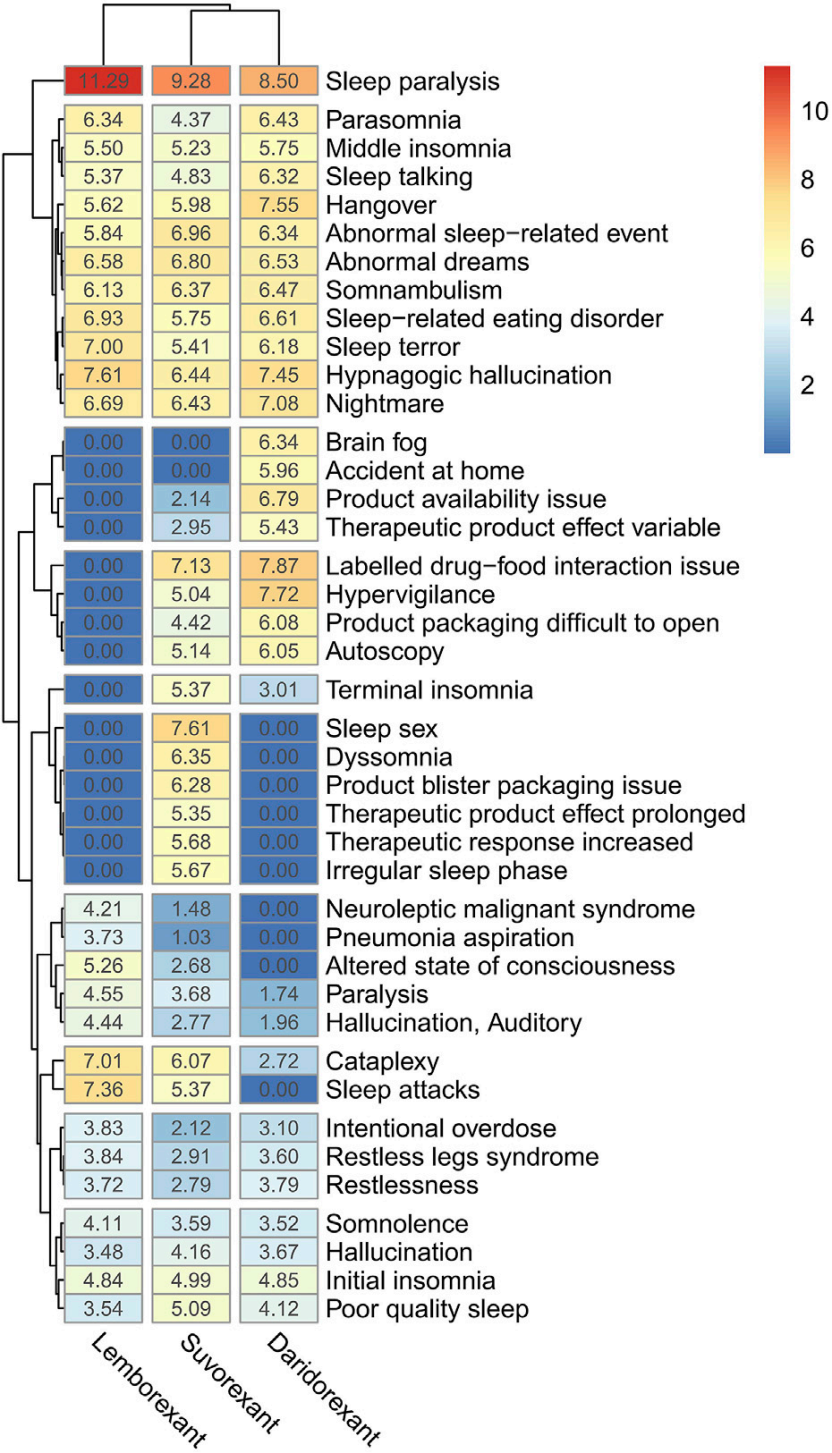
Clustering heatmap of differences in PT signals.

By taking the intersection of the top 20 PTs of the ROR signal intensity of three DORAs, a total of 41 PTs were obtained. The logarithm of the ROR value (+1) and draw a clustering heat map was calculated to compare the differences between the three drugs (Figure 3). Of these, “sleep paralysis” showed strong signals in all three DORAs. However, there are also differences between the three DORAs. For example, “brain fog” has a stronger signal in daridorexant, but was not detected for the other two drugs, whereas “sleep paralysis” and “dyssomnia” were stronger following suvorexant treatment but were also not detected for the other two drugs.

### 3.6 Differences between four data mining algorithms

To compare the differences between the four data mining algorithms, 1,251 DORA-related PTs were analysed. There are a total of 365 PTs that meet the requirements of at least one signal detection method, and 128 PTs simultaneously satisfied the four algorithms (As shown in Supplementary Table S1). The number of positive PTs that met the ROR, PRR, BCPNN, and MGPS were 214, 188297 and 199, respectively.

## 4 Discussion

Although drugs undergo rigorous clinical trials before being marketed, the adverse reactions that can occur during real-life practical application are not always completely predictable. Ongoing post-marketing research and monitoring can help identify and respond to these adverse effects in a timely manner. Using the FAERS database, this study performed signal mining and evaluation of DORAs, providing important information for future drug safety supervision.

Overall, the ADEs of the three DORAs evaluated were reported in a higher proportion of females than in males (57.27% vs. 33.04%), which is consistent with the higher incidence rate of insomnia in females reported in the literature, and is likely related to the complex interaction between biological, psychological, and social factors (Feinsilver, 2021; Carvalhas-Almeida et al., 2022; Tandon et al., 2022). Among reporting countries, the United States had the highest proportion compared to other regions, mainly because these drugs were first approved by the Food and Drug Administration, which is associated with to the FAERS system (the source of reports).

In this study, positive PT signals were detected based on ROR, PRR, MPGS, and BCPNN, and the number of SOCs involved were 14, 7, and 11, respectively (Table 3). After analyzing the SOC classification distribution, the terms “general disorders and administration site conditions,” “psychiatric disorders,” and “nervous system disorders” all ranked among the top three in terms of frequency for the three DORAs. In particular, a previous study found that suvorexant was associated with a seemingly dose-dependent worsening of depression and suicidal ideation (Petrous and Furmaga, 2017), and insomnia is also known as one of the main symptoms of depression (Nutt et al., 2008). Additionally, a study also found that daridorexant is more likely to cause depression (daridorexant vs all other drugs, ROR 2.13, daridorexant vs other DORAs, ROR: 2.31) (Cicala et al., 2024). Therefore, it is important to be alert to the possibility of associated events to avoid the possibility of worsening depression due to inadequate control of these ADEs.

In this study, using a combination of four signal detection methods (Table 1), the top 20 positive PT signals for suvorexant, lemborexant, daridorexant are listed in Tables 4–7. For suvorexant, lemborexant, and daridorexant, the highest signal strength was “sleep paralysis”. Sleep paralysis (SP) is a parasomnia characterised by a temporary immobility that occurs during sleep onset or upon awakening (Sateia, 2014). Sometimes they are accompanied by episodes of extreme fear reactions, hypnagogic and hypnopompic hallucinations (i.e., seeing, hearing, and feeling things that are not there) (D'Agostino and Limosani, 2016). Most prevalence studies suggest that 15%–40% of the population of younger individuals have experienced at least one episode of sleep paralysis (Stefani and Högl, 2021). Additionally, the signal intensity of “nightmare” was not the strongest among the three DORAs, but overall, it was the most frequent PT. The appearance of nightmares could be linked to the activity of DORAs as a class exerted on the global sleep architecture of patients, as DORAs can promote the REM phase of sleep. This increase in REM sleep time could facilitate the recall of dream content and its recall by the patient (Clark et al., 2020; Dal Sacco, 2022). A study reported that 24.9% of a sample of individuals with above-average psychopathology scores had clinically significant nightmare symptoms; however, 62.2% had not discussed their symptoms with a healthcare provider. This indicates that nightmares are, in general, often underreported and therefore, undetected (Nadorff et al., 2015). Since nightmares in patients with sleep disorders are also associated with increased levels of distress, this ADE should not be underestimated (Paul et al., 2015). Compared with traditional benzodiazepines and non-benzodiazepine drugs, DORAs inhibit the hyperactive arousal pathway in patients with insomnia by blocking orexin function (Beuckmann et al., 2017; Xue et al., 2023). This mechanism helps avoid side effects that can be caused by traditional insomnia medications, such as hangover. However, the PT of hangover was present in the first 20 positive PT signals of all three DORAs. Therefore, caution is warranted in clinical practice and patients should be informed of any adverse reactions when prescribing medication for insomnia, including potential effects on driving or other activities that require high attention the day following treatment (Wu et al., 2022).

After the intersection of the top 20 PTs of the ROR signal intensity for the three drugs, a clustering heatmap was drawn to visualize the differences between the different PTs of the three DORAs more intuitively (Figure 3), and “sleep paralysis” was detected as a strong signal in all DORAs. This PT signal was highest in lemborexant and lowest in daridorexant, consistent with the results of a recently published meta-study (Na et al., 2024). The reason for this may be related to the difference in half-life of the three drugs, with daridorexant having the shortest half-life (8 h) and lemborexant having the longest half-life (17–19 h) (Preskorn, 2022). Sleep paralysis may be caused by a selective loss or dysfunction of orexin (hypocretin) neurons in the lateral hypothalamus and is more likely to occur in patients with accompanying psychological disorders (Na et al., 2024). Sleep paralysis generally does not inflict physical harm, but approximately 90% of those who endure it grapple with fear. Furthermore, “brain fog” was stronger following daridorexant treatment but was not observed following treatment with the other two drugs, and “sleep sex” and “dyssomnia” were stronger with suvorexant but were not detected for the other two drugs. Additionally, when switching from other insomnia medication to suvorexant treatment, it is necessary to carefully monitor insomnia-related ADRs, which might be due to abrupt discontinuation of the prior insomnia medication use (Sano et al., 2019). Therefore, the differences in the PTs of the three DORAs should be noted when prescribing the DORAs, the situation of switching medication treatment and appropriate medication instructions should be provided in order to avoid these adverse effects as much as possible.

A study has found coherent between DORA and Z-drugs regarding suicidal ideation, but in terms of suicidal behavior, in comparison with Z-drugs, there were a relative paucity of cases of voluntary intoxication with DORA (Salvo et al., 2023). In this study, it was found that suicide was also a positive signal, but the intensity was relatively low (suicidal behaviour, ROR 5.95; suicidal ideationm, ROR: 3.49; suicide attempt, ROR: 2.66). Additionally, this study also found that some PTs related to DORAs use, such as “hangover,” “hypnagogic hallucination,” “middle insomnia,” “somnambulism,” or “sleep terror,” were not mentioned in the drug labels (For current labeling information, please visit https://www.fda.gov/drugsatfda). This suggests that the package insert may need to be further refined to include more comprehensive information on adverse reactions. Although some PTs are relatively rare, such as “hypnagogic hallucinations” and “hangover,” they ranked high in terms of signal intensity, suggesting that these new, rare, but potential ADRs cannot be ignored.

This study reveals the possible risks of DORAs for the treatment of insomnia. However, this study had some limitations. First, the ADEs recorded in the FAERS database were essentially spontaneous and most of the ADEs of DORAs derived from consumers, so there may be reporting biases (such as the impact of the disease itself, concomitant medications, *etc.*). Although this study identified PTs associated with the use of DORAs, this does not mean that these events were solely caused by these drugs and may be influenced by various factors, including the baseline characteristics of the patient, comorbidities, and concomitant medications. Therefore, this study used a variety of computational methods for signal mining. Furthermore, more prospective studies are still needed and evaluated in the context of actual clinical conditions to reduce the impact of these biases on the data.

## 5 Conclusion

In this study, four algorithms (ROR, PRR, BCPNN, and MGPS) were used to mine the safety signals of DORAs. A series of new potential PT signals, such as “hangover,” “hypnagogic hallucination,” “middle insomnia,” “somnambulism,” and “sleep terror,” were successfully identified. These signals are of great significance for guiding the safety of clinical drugs and for helping to improve the safety profile of DORAs. Future studies should combine other data sources and clinical trials to comprehensively evaluate the safety of DORAs.

## Data Availability

The raw data supporting the conclusions of this article will be made available by the authors, without undue reservation.
