# Supplementary material for: Data mining and safety analysis of dual orexin receptor antagonists (DORAs): a real-world pharmacovigilance study based on the FAERS database
Source: Front Pharmacol. 2024 Aug 6;15:1436405. doi: 10.3389/fphar.2024.1436405 (PMC11333359; doi:10.3389/fphar.2024.1436405)
Supplement: Supplementary file 1 [file Table1.DOCX]

**Table S1** The relevant PTs of DORAs meet the positive criteria of at least one data mining algorithm.

| **PTs** | **Frequency** | **ROR (95% CI)** | | **PRR (χ2)** | | **EBGM (EBGM05)** | | **IC (IC025)** | |
| --- | --- | --- | --- | --- | --- | --- | --- | --- | --- |
|  |  | **Value** | **Positive** | **Value** | **Positive** | **Value** | **Positive** | **Value** | **Positive** |
| Urine barbiturates | 1 | 1556.35 (97.34-24884.16) | No | 1556.29 (777.14) | No | 778.64 (76.57) | Yes | 9.60 (7.25) | Yes |
| Catathrenia | 2 | 1037.61 (173.37-6210.19) | No | 1037.53 (1242.63) | No | 622.92 (139.39) | Yes | 9.28 (7.24) | Yes |
| Sleep paralysis | 347 | 789.30 (693.44-898.42) | Yes | 778.14 (179553.23) | Yes | 519.10 (465.80) | Yes | 9.02 (7.35) | Yes |
| Tooth ankylosis | 1 | 311.27 (36.36-2664.50) | No | 311.26 (257.72) | No | 259.55 (43.05) | Yes | 8.02 (6.03) | Yes |
| Computerised tomogram head | 1 | 222.34 (27.35-1807.24) | No | 222.33 (192.79) | No | 194.66 (33.72) | Yes | 7.60 (5.68) | Yes |
| Exploding head syndrome | 2 | 172.93 (40.12-745.36) | No | 172.92 (307.67) | No | 155.73 (45.86) | Yes | 7.28 (5.50) | Yes |
| Labelled drug-food interaction issue | 8 | 163.87 (79.08-339.58) | Yes | 163.82 (1171.31) | Yes | 148.31 (80.61) | Yes | 7.21 (5.52) | Yes |
| Pseudoparalysis | 2 | 148.23 (34.75-632.22) | No | 148.22 (267.02) | No | 135.42 (40.23) | Yes | 7.08 (5.31) | Yes |
| Sleep sex | 3 | 137.34 (42.18-447.19) | Yes | 137.32 (373.06) | Yes | 126.27 (47.02) | Yes | 6.98 (5.25) | Yes |
| Hyposomnia | 2 | 135.34 (31.91-574.09) | No | 135.33 (245.34) | No | 124.58 (37.18) | Yes | 6.96 (5.20) | Yes |
| Limited symptom panic attack | 1 | 119.72 (15.66-915.23) | No | 119.71 (109.31) | No | 111.23 (20.28) | Yes | 6.80 (4.97) | Yes |
| Manufacturing equipment issue | 1 | 119.72 (15.66-915.23) | No | 119.71 (109.31) | No | 111.23 (20.28) | Yes | 6.80 (4.97) | Yes |
| Hypnagogic hallucination | 13 | 117.01 (66.58-205.62) | Yes | 116.95 (1389.97) | Yes | 108.84 (67.91) | Yes | 6.77 (5.09) | Yes |
| Abnormal sleep-related event | 43 | 112.48 (82.52-153.32) | Yes | 112.28 (4423.47) | Yes | 104.79 (80.87) | Yes | 6.71 (5.04) | Yes |
| Traumatic tooth displacement | 1 | 111.17 (14.62-845.45) | No | 111.16 (101.89) | No | 103.82 (19.01) | Yes | 6.70 (4.88) | Yes |
| Abnormal dreams | 789 | 107.57 (99.98-115.74) | Yes | 104.14 (75565.88) | Yes | 97.67 (91.87) | Yes | 6.61 (4.94) | Yes |
| Nightmare | 1145 | 100.80 (94.83-107.14) | Yes | 96.13 (101577.02) | Yes | 90.60 (86.09) | Yes | 6.50 (4.84) | Yes |
| Hangover | 129 | 94.97 (79.47-113.49) | Yes | 94.48 (11248.60) | Yes | 89.13 (76.78) | Yes | 6.48 (4.81) | Yes |
| Somnambulism | 188 | 84.50 (72.93-97.91) | Yes | 83.86 (14606.09) | Yes | 79.62 (70.39) | Yes | 6.32 (4.65) | Yes |
| Hypervigilance | 26 | 76.14 (51.35-112.89) | Yes | 76.06 (1836.17) | Yes | 72.56 (52.19) | Yes | 6.18 (4.51) | Yes |
| Sleep-related eating disorder | 25 | 68.09 (45.61-101.64) | Yes | 68.02 (1581.75) | Yes | 65.21 (46.64) | Yes | 6.03 (4.36) | Yes |
| Hypnopompic hallucination | 3 | 64.85 (20.43-205.85) | Yes | 64.85 (181.04) | Yes | 62.29 (23.70) | Yes | 5.96 (4.26) | Yes |
| Antidepressant therapy | 1 | 62.25 (8.43-459.47) | No | 62.25 (57.95) | No | 59.9 (11.25) | Yes | 5.90 (4.15) | Yes |
| Caffeine consumption | 1 | 59.86 (8.12-441.15) | No | 59.86 (55.73) | No | 57.68 (10.84) | Yes | 5.85 (4.10) | Yes |
| Dyssomnia | 4 | 57.12 (21.06-154.94) | Yes | 57.11 (212.71) | Yes | 55.13 (23.92) | Yes | 5.78 (4.10) | Yes |
| Vaginal oedema | 1 | 55.58 (7.56-408.56) | No | 55.58 (51.75) | No | 53.70 (10.12) | Yes | 5.75 (4.00) | Yes |
| Product blister packaging issue | 26 | 54.44 (36.82-80.51) | Yes | 54.39 (1316.54) | Yes | 52.58 (37.90) | Yes | 5.72 (4.05) | Yes |
| Cataplexy | 38 | 54.24 (39.24-74.97) | Yes | 54.16 (1916.03) | Yes | 52.37 (39.94) | Yes | 5.71 (4.04) | Yes |
| Sleep terror | 86 | 53.74 (43.33-66.66) | Yes | 53.56 (4288.30) | Yes | 51.81 (43.27) | Yes | 5.70 (4.03) | Yes |
| Barbiturates positive | 1 | 51.88 (7.07-380.44) | No | 51.88 (48.29) | No | 50.24 (9.48) | Yes | 5.65 (3.91) | Yes |
| Phobia of driving | 3 | 49.15 (15.57-155.14) | Yes | 49.15 (137.17) | Yes | 47.67 (18.22) | Yes | 5.58 (3.88) | Yes |
| Exhibitionism | 1 | 48.64 (6.65-355.95) | No | 48.63 (45.24) | No | 47.19 (8.92) | Yes | 5.56 (3.82) | Yes |
| Poriomania | 2 | 45.78 (11.22-186.79) | No | 45.77 (85.09) | No | 44.49 (13.72) | Yes | 5.48 (3.77) | Yes |
| Middle insomnia | 253 | 41.39 (36.51-46.93) | Yes | 40.98 (9616.52) | Yes | 39.95 (35.97) | Yes | 5.32 (3.65) | Yes |
| Sleep talking | 23 | 41.28 (27.28-62.46) | Yes | 41.24 (879.74) | Yes | 40.20 (28.42) | Yes | 5.33 (3.66) | Yes |
| Autoscopy | 10 | 40.97 (21.86-76.78) | Yes | 40.95 (379.80) | Yes | 39.93 (23.61) | Yes | 5.32 (3.65) | Yes |
| Parasomnia | 11 | 39.01 (21.44-70.98) | Yes | 39.00 (397.29) | Yes | 38.07 (23.07) | Yes | 5.25 (3.58) | Yes |
| Sleep attacks | 5 | 36.03 (14.85-87.46) | Yes | 36.03 (166.41) | Yes | 35.23 (16.78) | Yes | 5.14 (3.46) | Yes |
| Loss of dreaming | 2 | 35.78 (8.81-145.36) | No | 35.78 (66.09) | No | 35.00 (10.83) | Yes | 5.13 (3.44) | Yes |
| Therapeutic response increased | 13 | 35.51 (20.49-61.55) | Yes | 35.49 (426.08) | Yes | 34.73 (21.92) | Yes | 5.12 (3.45) | Yes |
| Irregular sleep phase | 3 | 35.37 (11.26-111.10) | Yes | 35.37 (97.97) | Yes | 34.61 (13.28) | Yes | 5.11 (3.43) | Yes |
| Glossoptosis | 2 | 33.47 (8.25-135.84) | No | 33.47 (61.67) | No | 32.79 (10.15) | Yes | 5.03 (3.34) | Yes |
| Amputee | 1 | 32.42 (4.48-234.92) | No | 32.42 (29.83) | No | 31.78 (6.06) | Yes | 4.99 (3.27) | Yes |
| Product packaging difficult to open | 30 | 31.27 (21.78-44.89) | Yes | 31.23 (860.62) | Yes | 30.64 (22.64) | Yes | 4.94 (3.27) | Yes |
| Terminal insomnia | 17 | 30.33 (18.76-49.02) | Yes | 30.31 (472.57) | Yes | 29.75 (19.90) | Yes | 4.89 (3.23) | Yes |
| Initial insomnia | 99 | 30.19 (24.74-36.85) | Yes | 30.07 (2730.30) | Yes | 29.52 (24.99) | Yes | 4.88 (3.22) | Yes |
| Taciturnity | 1 | 29.37 (4.06-212.35) | No | 29.36 (26.89) | No | 28.84 (5.51) | Yes | 4.85 (3.14) | Yes |
| Product availability issue | 256 | 28.79 (25.43-32.60) | Yes | 28.50 (6673.96) | Yes | 28.01 (25.24) | Yes | 4.81 (3.14) | Yes |
| Therapeutic product effect prolonged | 10 | 28.20 (15.09-52.72) | Yes | 28.19 (257.63) | Yes | 27.71 (16.42) | Yes | 4.79 (3.12) | Yes |
| Poor quality sleep | 238 | 28.09 (24.70-31.96) | Yes | 27.83 (6050.21) | Yes | 27.36 (24.56) | Yes | 4.77 (3.11) | Yes |
| Visual perseveration | 2 | 27.31 (6.75-110.52) | No | 27.30 (49.81) | No | 26.85 (8.33) | Yes | 4.75 (3.06) | Yes |
| Interventional procedure | 1 | 25.94 (3.59-187.18) | No | 25.94 (23.58) | No | 25.53 (4.89) | Yes | 4.67 (2.97) | Yes |
| Alcoholic | 2 | 25.31 (6.26-102.34) | No | 25.31 (45.94) | No | 24.92 (7.74) | Yes | 4.64 (2.95) | Yes |
| Labelled drug-food interaction medication error | 6 | 24.64 (11.00-55.21) | Yes | 24.64 (133.95) | Yes | 24.27 (12.36) | Yes | 4.60 (2.93) | Yes |
| Ebstein's anomaly | 1 | 24.32 (3.37-175.30) | No | 24.32 (22.01) | No | 23.96 (4.59) | Yes | 4.58 (2.88) | Yes |
| Tachyphrenia | 31 | 24.09 (16.89-34.36) | Yes | 24.06 (674.82) | Yes | 23.71 (17.62) | Yes | 4.57 (2.90) | Yes |
| Paraphilia | 1 | 23.58 (3.27-169.91) | No | 23.58 (21.30) | No | 23.24 (4.45) | Yes | 4.54 (2.84) | Yes |
| Spontaneous penile erection | 5 | 23.51 (9.72-56.87) | Yes | 23.51 (106.15) | Yes | 23.17 (11.07) | Yes | 4.53 (2.86) | Yes |
| Therapeutic product effect delayed | 91 | 22.15 (18.00-27.25) | Yes | 22.07 (1805.19) | Yes | 21.78 (18.31) | Yes | 4.44 (2.78) | Yes |
| Dreamy state | 2 | 20.21 (5.01-81.56) | No | 20.21 (36.05) | No | 19.97 (6.21) | Yes | 4.32 (2.64) | Yes |
| Waxy flexibility | 1 | 19.95 (2.78-143.45) | No | 19.95 (17.77) | No | 19.71 (3.78) | Yes | 4.30 (2.60) | Yes |
| Brain fog | 44 | 19.72 (14.64-26.55) | Yes | 19.68 (770.61) | Yes | 19.45 (15.16) | Yes | 4.28 (2.61) | Yes |
| Somnolence neonatal | 1 | 19.45 (2.71-139.82) | No | 19.45 (17.29) | No | 19.23 (3.69) | Yes | 4.26 (2.57) | Yes |
| Packaging design issue | 3 | 19.14 (6.13-59.75) | Yes | 19.13 (50.93) | Yes | 18.91 (7.30) | Yes | 4.24 (2.57) | Yes |
| Product compounding quality issue | 2 | 19.10 (4.74-77.01) | No | 19.10 (33.88) | No | 18.88 (5.88) | Yes | 4.24 (2.56) | Yes |
| Manufacturing production issue | 1 | 18.75 (2.61-134.70) | No | 18.75 (16.60) | No | 18.54 (3.56) | Yes | 4.21 (2.52) | Yes |
| Alcohol test false positive | 1 | 18.53 (2.58-133.08) | No | 18.53 (16.39) | No | 18.32 (3.52) | Yes | 4.20 (2.50) | Yes |
| Dissociative disorder | 4 | 17.59 (6.56-47.13) | Yes | 17.59 (61.87) | Yes | 17.40 (7.63) | Yes | 4.12 (2.45) | Yes |
| Circadian rhythm sleep disorder | 5 | 17.18 (7.12-41.48) | Yes | 17.18 (75.35) | Yes | 17.00 (8.13) | Yes | 4.09 (2.42) | Yes |
| Pharyngeal neoplasm | 1 | 17.10 (2.38-122.73) | No | 17.10 (15.00) | No | 16.93 (3.25) | Yes | 4.08 (2.39) | Yes |
| Anterograde amnesia | 4 | 17.01 (6.35-45.57) | Yes | 17.01 (59.62) | Yes | 16.84 (7.38) | Yes | 4.07 (2.40) | Yes |
| Product dosage form confusion | 1 | 16.92 (2.36-121.39) | No | 16.92 (14.81) | No | 16.75 (3.22) | Yes | 4.07 (2.37) | Yes |
| Product packaging quantity issue | 69 | 16.43 (12.95-20.83) | Yes | 16.38 (986.53) | Yes | 16.22 (13.30) | Yes | 4.02 (2.35) | Yes |
| Gastric fistula | 3 | 15.94 (5.11-49.70) | Yes | 15.93 (41.57) | Yes | 15.78 (6.09) | Yes | 3.98 (2.31) | Yes |
| Psychotic disorder due to a general medical condition | 1 | 15.56 (2.17-111.58) | No | 15.56 (13.49) | No | 15.42 (2.97) | Yes | 3.95 (2.26) | Yes |
| Hallucination | 412 | 15.36 (13.93-16.94) | Yes | 15.12 (5386) | Yes | 14.98 (13.81) | Yes | 3.91 (2.24) | Yes |
| Therapeutic product effect variable | 38 | 15.26 (11.09-21.02) | Yes | 15.24 (500.84) | Yes | 15.10 (11.56) | Yes | 3.92 (2.25) | Yes |
| Paralysis recurrent laryngeal nerve | 1 | 15.26 (2.13-109.37) | No | 15.26 (13.19) | No | 15.12 (2.91) | Yes | 3.92 (2.23) | Yes |
| Accident at home | 3 | 15.11 (4.85-47.12) | Yes | 15.11 (39.15) | Yes | 14.97 (5.78) | Yes | 3.90 (2.23) | Yes |
| Antisynthetase syndrome | 1 | 15.11 (2.11-108.3) | No | 15.11 (13.05) | No | 14.97 (2.88) | Yes | 3.90 (2.21) | Yes |
| Therapeutic response delayed | 15 | 14.84 (8.92-24.68) | Yes | 14.83 (191.66) | Yes | 14.70 (9.61) | Yes | 3.88 (2.21) | Yes |
| Confusional arousal | 1 | 14.82 (2.07-106.22) | No | 14.82 (12.77) | No | 14.69 (2.83) | Yes | 3.88 (2.19) | Yes |
| Strangury | 1 | 14.82 (2.07-106.22) | No | 14.82 (12.77) | No | 14.69 (2.83) | Yes | 3.88 (2.19) | Yes |
| Exaggerated startle response | 1 | 14.41 (2.01-103.24) | No | 14.41 (12.37) | No | 14.29 (2.75) | Yes | 3.84 (2.15) | Yes |
| Screaming | 28 | 14.19 (9.78-20.59) | Yes | 14.18 (339.83) | Yes | 14.06 (10.29) | Yes | 3.81 (2.15) | Yes |
| Rapid eye movements sleep abnormal | 2 | 14.09 (3.50-56.68) | No | 14.08 (24.09) | No | 13.97 (4.36) | Yes | 3.80 (2.13) | Yes |
| Sudden onset of sleep | 6 | 13.74 (6.15-30.68) | Yes | 13.73 (70.21) | Yes | 13.62 (6.95) | Yes | 3.77 (2.10) | Yes |
| Intraocular pressure test | 1 | 13.53 (1.89-96.9) | No | 13.53 (11.51) | No | 13.42 (2.59) | Yes | 3.75 (2.06) | Yes |
| Gene mutation identification test positive | 2 | 13.48 (3.35-54.21) | No | 13.47 (22.90) | No | 13.37 (4.17) | Yes | 3.74 (2.06) | Yes |
| Retrograde amnesia | 2 | 13.08 (3.25-52.60) | No | 13.08 (22.12) | No | 12.98 (4.05) | Yes | 3.70 (2.02) | Yes |
| Narcolepsy | 10 | 13.04 (7.00-24.3) | Yes | 13.03 (110.19) | Yes | 12.93 (7.68) | Yes | 3.69 (2.03) | Yes |
| Legal problem | 2 | 12.71 (3.16-51.09) | No | 12.70 (21.39) | No | 12.61 (3.94) | Yes | 3.66 (1.98) | Yes |
| Oscillopsia | 1 | 12.65 (1.77-90.55) | No | 12.65 (10.65) | No | 12.56 (2.42) | Yes | 3.65 (1.96) | Yes |
| Rapid eye movement sleep behaviour disorder | 2 | 12.45 (3.10-50.07) | No | 12.45 (20.89) | No | 12.36 (3.86) | Yes | 3.63 (1.95) | Yes |
| Pituitary enlargement | 1 | 12.45 (1.74-89.09) | No | 12.45 (10.45) | No | 12.36 (2.38) | Yes | 3.63 (1.94) | Yes |
| Flashback | 2 | 12.40 (3.08-49.87) | No | 12.40 (20.8) | No | 12.31 (3.84) | Yes | 3.62 (1.95) | Yes |
| Suicide threat | 2 | 12.16 (3.02-48.89) | No | 12.16 (20.32) | No | 12.07 (3.77) | Yes | 3.59 (1.92) | Yes |
| Intracranial hypotension | 1 | 12.06 (1.69-86.31) | No | 12.06 (10.07) | No | 11.98 (2.31) | Yes | 3.58 (1.90) | Yes |
| Hand-eye coordination impaired | 1 | 11.70 (1.64-83.69) | No | 11.70 (9.71) | No | 11.62 (2.24) | Yes | 3.54 (1.85) | Yes |
| Product outer packaging issue | 4 | 11.36 (4.25-30.38) | Yes | 11.36 (37.52) | Yes | 11.28 (4.95) | Yes | 3.50 (1.83) | Yes |
| Disturbance in sexual arousal | 4 | 11.34 (4.24-30.33) | Yes | 11.34 (37.44) | Yes | 11.26 (4.95) | Yes | 3.49 (1.82) | Yes |
| Somnolence | 831 | 11.16 (10.41-11.96) | Yes | 10.82 (7376.17) | Yes | 10.75 (10.14) | Yes | 3.43 (1.76) | Yes |
| Time perception altered | 2 | 11.04 (2.75-44.36) | No | 11.04 (18.13) | No | 10.97 (3.42) | Yes | 3.46 (1.78) | Yes |
| Vibratory sense increased | 1 | 11.04 (1.54-78.91) | No | 11.04 (9.06) | No | 10.97 (2.11) | Yes | 3.46 (1.77) | Yes |
| Feeling jittery | 73 | 10.83 (8.60-13.63) | Yes | 10.80 (644.74) | Yes | 10.73 (8.85) | Yes | 3.42 (1.76) | Yes |
| Chromatopsia | 2 | 10.81 (2.69-43.43) | No | 10.81 (17.68) | No | 10.74 (3.35) | Yes | 3.42 (1.75) | Yes |
| Product quality control issue | 1 | 10.66 (1.49-76.19) | No | 10.66 (8.69) | No | 10.59 (2.04) | Yes | 3.41 (1.72) | Yes |
| Head banging | 2 | 10.45 (2.60-41.96) | No | 10.44 (16.97) | No | 10.38 (3.24) | Yes | 3.38 (1.70) | Yes |
| Dyscalculia | 1 | 10.31 (1.44-73.65) | No | 10.31 (8.35) | No | 10.25 (1.98) | No | 3.36 (1.67) | Yes |
| Claustrophobia | 4 | 10.24 (3.83-27.37) | Yes | 10.24 (33.13) | Yes | 10.18 (4.47) | Yes | 3.35 (1.68) | Yes |
| Labelled drug-drug interaction issue | 4 | 10.09 (3.77-26.97) | Yes | 10.09 (32.54) | Yes | 10.03 (4.41) | Yes | 3.33 (1.66) | Yes |
| Opiates positive | 2 | 9.95 (2.48-39.94) | No | 9.94 (15.99) | No | 9.89 (3.09) | Yes | 3.31 (1.63) | Yes |
| Paralysis | 51 | 9.94 (7.55-13.09) | Yes | 9.92 (406.62) | Yes | 9.86 (7.83) | Yes | 3.30 (1.64) | Yes |
| Arrhythmic storm | 1 | 9.85 (1.38-70.37) | No | 9.85 (7.90) | No | 9.79 (1.89) | No | 3.29 (1.61) | Yes |
| Meningeal disorder | 1 | 9.61 (1.35-68.62) | No | 9.61 (7.66) | No | 9.55 (1.84) | No | 3.26 (1.57) | Yes |
| Limb traumatic amputation | 1 | 9.55 (1.34-68.20) | No | 9.55 (7.61) | No | 9.50 (1.83) | No | 3.25 (1.57) | Yes |
| Gallbladder obstruction | 1 | 9.43 (1.32-67.36) | No | 9.43 (7.49) | No | 9.38 (1.81) | No | 3.23 (1.55) | Yes |
| Suspected product tampering | 2 | 9.38 (2.33-37.65) | No | 9.38 (14.87) | No | 9.33 (2.91) | Yes | 3.22 (1.55) | Yes |
| Enuresis | 14 | 9.32 (5.51-15.77) | Yes | 9.32 (103.36) | Yes | 9.27 (5.97) | Yes | 3.21 (1.55) | Yes |
| Mitral valve repair | 1 | 9.32 (1.30-66.55) | No | 9.32 (7.38) | No | 9.27 (1.79) | No | 3.21 (1.53) | Yes |
| Feeling drunk | 25 | 9.21 (6.22-13.65) | Yes | 9.20 (181.72) | Yes | 9.15 (6.59) | Yes | 3.19 (1.53) | Yes |
| Daydreaming | 6 | 9.19 (4.12-20.51) | Yes | 9.19 (43.54) | Yes | 9.14 (4.67) | Yes | 3.19 (1.52) | Yes |
| Sudden visual loss | 1 | 9.10 (1.27-64.99) | No | 9.10 (7.17) | No | 9.05 (1.75) | No | 3.18 (1.50) | Yes |
| Breathing-related sleep disorder | 1 | 9.05 (1.27-64.61) | No | 9.05 (7.12) | No | 9.00 (1.74) | No | 3.17 (1.49) | Yes |
| Electrocardiogram qrs complex abnormal | 1 | 8.89 (1.25-63.49) | No | 8.89 (6.97) | No | 8.85 (1.71) | No | 3.15 (1.47) | Yes |
| Pertussis | 3 | 8.81 (2.83-27.41) | Yes | 8.81 (20.65) | Yes | 8.77 (3.39) | Yes | 3.13 (1.46) | Yes |
| Oral discharge | 1 | 8.79 (1.23-62.77) | No | 8.79 (6.87) | No | 8.75 (1.69) | No | 3.13 (1.45) | Yes |
| Diplegia | 6 | 8.75 (3.92-19.51) | Yes | 8.74 (40.92) | Yes | 8.70 (4.45) | Yes | 3.12 (1.45) | Yes |
| Trance | 1 | 8.60 (1.20-61.38) | No | 8.60 (6.68) | No | 8.56 (1.65) | No | 3.10 (1.42) | Yes |
| Insomnia | 843 | 8.54 (7.97-9.14) | Yes | 8.28 (5386.79) | Yes | 8.24 (7.78) | Yes | 3.04 (1.38) | Yes |
| Malignant neoplasm of eye | 1 | 8.50 (1.19-60.7) | No | 8.50 (6.59) | No | 8.46 (1.63) | No | 3.08 (1.40) | Yes |
| Fracture displacement | 1 | 8.23 (1.15-58.77) | No | 8.23 (6.32) | No | 8.20 (1.58) | No | 3.03 (1.36) | Yes |
| Delirium | 101 | 8.15 (6.70-9.91) | Yes | 8.12 (627.35) | Yes | 8.08 (6.86) | Yes | 3.01 (1.35) | Yes |
| Patient isolation | 1 | 7.98 (1.12-56.95) | No | 7.98 (6.08) | No | 7.95 (1.53) | No | 2.99 (1.31) | Yes |
| Restless legs syndrome | 55 | 7.97 (6.11-10.39) | Yes | 7.95 (332.79) | Yes | 7.92 (6.34) | Yes | 2.99 (1.32) | Yes |
| Restlessness | 105 | 7.92 (6.53-9.59) | Yes | 7.89 (628.57) | Yes | 7.85 (6.69) | Yes | 2.97 (1.31) | Yes |
| Energy increased | 19 | 7.77 (4.95-12.20) | Yes | 7.77 (111.48) | Yes | 7.73 (5.30) | Yes | 2.95 (1.28) | Yes |
| Quarantine | 1 | 7.74 (1.09-55.24) | No | 7.74 (5.84) | No | 7.71 (1.49) | No | 2.95 (1.27) | Yes |
| Postrenal failure | 1 | 7.67 (1.07-54.69) | No | 7.67 (5.77) | No | 7.63 (1.47) | No | 2.93 (1.25) | Yes |
| Concomitant disease aggravated | 23 | 7.63 (5.06-11.50) | Yes | 7.62 (131.74) | Yes | 7.59 (5.39) | Yes | 2.92 (1.26) | Yes |
| Bipolar ii disorder | 1 | 7.59 (1.06-54.16) | No | 7.59 (5.70) | No | 7.56 (1.46) | No | 2.92 (1.24) | Yes |
| Oesophageal food impaction | 1 | 7.52 (1.05-53.63) | No | 7.52 (5.62) | No | 7.49 (1.45) | No | 2.90 (1.23) | Yes |
| Periodic limb movement disorder | 1 | 7.52 (1.05-53.63) | No | 7.52 (5.62) | No | 7.49 (1.45) | No | 2.90 (1.23) | Yes |
| Tonsillar inflammation | 1 | 7.52 (1.05-53.63) | No | 7.52 (5.62) | No | 7.49 (1.45) | No | 2.90 (1.23) | Yes |
| Atonic seizures | 2 | 7.43 (1.85-29.81) | No | 7.43 (11.07) | No | 7.40 (2.31) | Yes | 2.89 (1.22) | Yes |
| Gastrointestinal candidiasis | 1 | 7.38 (1.03-52.61) | No | 7.38 (5.49) | No | 7.35 (1.42) | No | 2.88 (1.20) | Yes |
| Paradoxical drug reaction | 11 | 7.36 (4.07-13.30) | Yes | 7.35 (60.11) | Yes | 7.32 (4.46) | Yes | 2.87 (1.21) | Yes |
| Product shape issue | 3 | 7.31 (2.35-22.72) | Yes | 7.31 (16.25) | Yes | 7.28 (2.82) | Yes | 2.86 (1.19) | Yes |
| Orgasm abnormal | 2 | 7.14 (1.78-28.64) | No | 7.14 (10.51) | No | 7.11 (2.22) | Yes | 2.83 (1.16) | Yes |
| Drug effect faster than expected | 1 | 7.07 (0.99-50.45) | No | 7.07 (5.19) | No | 7.05 (1.36) | No | 2.82 (1.14) | Yes |
| Blast cell crisis | 1 | 7.04 (0.99-50.22) | No | 7.04 (5.16) | No | 7.01 (1.36) | No | 2.81 (1.13) | Yes |
| Thinking abnormal | 46 | 7.01 (5.25-9.37) | Yes | 7.00 (235.67) | Yes | 6.98 (5.47) | Yes | 2.80 (1.14) | Yes |
| Seizure like phenomena | 5 | 7.01 (2.91-16.88) | Yes | 7.01 (25.65) | Yes | 6.98 (3.35) | Yes | 2.80 (1.14) | Yes |
| Drug ineffective | 3493 | 6.95 (6.71-7.21) | Yes | 6.11 (15212.76) | Yes | 6.09 (5.91) | Yes | 2.61 (0.94) | Yes |
| Mouth breathing | 1 | 6.95 (0.97-49.54) | No | 6.95 (5.07) | No | 6.92 (1.34) | No | 2.79 (1.11) | Yes |
| Therapeutic product effect increased | 11 | 6.91 (3.82-12.49) | Yes | 6.91 (55.31) | Yes | 6.88 (4.19) | Yes | 2.78 (1.12) | Yes |
| Lack of spontaneous speech | 1 | 6.89 (0.97-49.10) | No | 6.89 (5.01) | No | 6.86 (1.33) | No | 2.78 (1.10) | Yes |
| Resting tremor | 2 | 6.87 (1.71-27.56) | No | 6.87 (9.99) | No | 6.85 (2.14) | Yes | 2.78 (1.10) | Yes |
| Sluggishness | 28 | 6.82 (4.70-9.88) | Yes | 6.81 (138.21) | Yes | 6.78 (4.97) | Yes | 2.76 (1.10) | Yes |
| Irregular sleep wake rhythm disorder | 1 | 6.80 (0.95-48.46) | No | 6.80 (4.92) | No | 6.77 (1.31) | No | 2.76 (1.08) | Yes |
| Nocturia | 31 | 6.79 (4.77-9.67) | Yes | 6.78 (152.23) | Yes | 6.76 (5.03) | Yes | 2.76 (1.09) | Yes |
| Sleep disorder | 189 | 6.77 (5.86-7.81) | Yes | 6.72 (917.72) | Yes | 6.70 (5.94) | Yes | 2.74 (1.08) | Yes |
| Inability to afford medication | 27 | 6.68 (4.57-9.74) | Yes | 6.67 (129.57) | Yes | 6.64 (4.84) | Yes | 2.73 (1.07) | Yes |
| Product after taste | 2 | 6.67 (1.66-26.73) | No | 6.67 (9.59) | No | 6.64 (2.08) | Yes | 2.73 (1.06) | Yes |
| Impaired driving ability | 24 | 6.61 (4.42-9.87) | Yes | 6.60 (113.58) | Yes | 6.58 (4.70) | Yes | 2.72 (1.05) | Yes |
| Product container seal issue | 4 | 6.60 (2.47-17.63) | Yes | 6.60 (18.93) | Yes | 6.58 (2.89) | Yes | 2.72 (1.05) | Yes |
| Loss of therapeutic response | 6 | 6.41 (2.87-14.29) | Yes | 6.41 (27.28) | Yes | 6.39 (3.26) | Yes | 2.68 (1.01) | Yes |
| Intentional underdose | 24 | 6.24 (4.18-9.32) | Yes | 6.23 (105.05) | Yes | 6.21 (4.44) | Yes | 2.64 (0.97) | Yes |
| Agitation | 157 | 6.23 (5.32-7.29) | Yes | 6.20 (682.24) | Yes | 6.18 (5.41) | Yes | 2.63 (0.96) | Yes |
| Sticky skin | 1 | 6.23 (0.87-44.37) | No | 6.23 (4.37) | No | 6.20 (1.20) | No | 2.63 (0.96) | Yes |
| Nocturnal dyspnoea | 3 | 6.17 (1.98-19.17) | Yes | 6.17 (12.94) | Yes | 6.15 (2.38) | Yes | 2.62 (0.95) | Yes |
| Product substitution | 1 | 6.15 (0.86-43.84) | No | 6.15 (4.30) | No | 6.13 (1.19) | No | 2.62 (0.94) | Yes |
| Feeling abnormal | 593 | 6.11 (5.63-6.63) | Yes | 5.99 (2464.89) | Yes | 5.97 (5.58) | Yes | 2.58 (0.91) | Yes |
| Frontotemporal dementia | 1 | 5.99 (0.84-42.66) | No | 5.99 (4.14) | No | 5.97 (1.15) | No | 2.58 (0.90) | Yes |
| Formication | 15 | 5.98 (3.60-9.93) | Yes | 5.98 (61.97) | Yes | 5.96 (3.90) | Yes | 2.58 (0.91) | Yes |
| Product confusion | 2 | 5.97 (1.49-23.95) | No | 5.97 (8.25) | No | 5.96 (1.86) | No | 2.57 (0.90) | Yes |
| Fear | 49 | 5.96 (4.50-7.89) | Yes | 5.95 (200.96) | Yes | 5.93 (4.69) | Yes | 2.57 (0.90) | Yes |
| Suicidal behaviour | 9 | 5.95 (3.09-11.46) | Yes | 5.95 (36.95) | Yes | 5.93 (3.43) | Yes | 2.57 (0.90) | Yes |
| Morbid thoughts | 2 | 5.83 (1.45-23.37) | No | 5.83 (7.97) | No | 5.81 (1.82) | No | 2.54 (0.87) | Yes |
| Drug tolerance increased | 2 | 5.83 (1.45-23.37) | No | 5.83 (7.97) | No | 5.81 (1.82) | No | 2.54 (0.87) | Yes |
| Open fracture | 1 | 5.83 (0.82-41.54) | No | 5.83 (3.99) | No | 5.81 (1.12) | No | 2.54 (0.86) | Yes |
| Stupor | 6 | 5.80 (2.60-12.93) | Yes | 5.80 (23.73) | Yes | 5.78 (2.95) | Yes | 2.53 (0.86) | Yes |
| Sleep deficit | 5 | 5.78 (2.40-13.91) | Yes | 5.78 (19.68) | Yes | 5.76 (2.76) | Yes | 2.53 (0.86) | Yes |
| Hallucination, auditory | 32 | 5.75 (4.06-8.13) | Yes | 5.74 (124.87) | Yes | 5.72 (4.28) | Yes | 2.52 (0.85) | Yes |
| Meniere's disease | 3 | 5.60 (1.80-17.40) | Yes | 5.60 (11.29) | Yes | 5.58 (2.16) | Yes | 2.48 (0.81) | Yes |
| Drug screen false positive | 5 | 5.59 (2.32-13.46) | Yes | 5.59 (18.78) | Yes | 5.57 (2.67) | Yes | 2.48 (0.81) | Yes |
| Incoherent | 11 | 5.54 (3.07-10.02) | Yes | 5.54 (40.80) | Yes | 5.53 (3.37) | Yes | 2.47 (0.80) | Yes |
| Adverse event | 202 | 5.48 (4.77-6.30) | Yes | 5.45 (732.09) | Yes | 5.43 (4.84) | Yes | 2.44 (0.78) | Yes |
| Altered state of consciousness | 44 | 5.43 (4.04-7.31) | Yes | 5.43 (158.37) | Yes | 5.41 (4.22) | Yes | 2.44 (0.77) | Yes |
| Psychomotor hyperactivity | 32 | 5.36 (3.79-7.59) | Yes | 5.35 (112.97) | Yes | 5.34 (3.99) | Yes | 2.42 (0.75) | Yes |
| Necrotising oesophagitis | 1 | 5.35 (0.75-38.10) | No | 5.35 (3.52) | No | 5.33 (1.03) | No | 2.41 (0.74) | Yes |
| Gun shot wound | 2 | 5.33 (1.33-21.36) | No | 5.33 (7.01) | No | 5.31 (1.66) | No | 2.41 (0.74) | Yes |
| Hypersomnia | 59 | 5.29 (4.10-6.83) | Yes | 5.28 (204.07) | Yes | 5.27 (4.25) | Yes | 2.40 (0.73) | Yes |
| Amnestic disorder | 1 | 5.29 (0.74-37.71) | No | 5.29 (3.47) | No | 5.28 (1.02) | No | 2.40 (0.73) | Yes |
| Tinea cruris | 1 | 5.28 (0.74-37.58) | No | 5.28 (3.45) | No | 5.26 (1.02) | No | 2.40 (0.72) | Yes |
| Panic reaction | 14 | 5.27 (3.12-8.90) | Yes | 5.26 (48.20) | Yes | 5.25 (3.38) | Yes | 2.39 (0.73) | Yes |
| Osteogenesis imperfecta | 1 | 5.15 (0.72-36.71) | No | 5.15 (3.34) | No | 5.14 (0.99) | No | 2.36 (0.69) | Yes |
| Sexually inappropriate behaviour | 1 | 5.12 (0.72-36.46) | No | 5.12 (3.30) | No | 5.11 (0.99) | No | 2.35 (0.68) | Yes |
| Thought blocking | 1 | 5.09 (0.71-36.22) | No | 5.09 (3.27) | No | 5.07 (0.98) | No | 2.34 (0.67) | Yes |
| Moaning | 2 | 5.06 (1.26-20.29) | No | 5.06 (6.50) | No | 5.05 (1.58) | No | 2.34 (0.67) | Yes |
| Therapeutic product effect incomplete | 282 | 5.05 (4.49-5.68) | Yes | 5.01 (902.98) | Yes | 4.99 (4.52) | Yes | 2.32 (0.65) | Yes |
| Brain stem stroke | 1 | 5.05 (0.71-35.99) | No | 5.05 (3.24) | No | 5.04 (0.97) | No | 2.33 (0.66) | Yes |
| Collagen disorder | 1 | 5.02 (0.70-35.76) | No | 5.02 (3.21) | No | 5.01 (0.97) | No | 2.32 (0.65) | Yes |
| Hypermetabolism | 1 | 5.00 (0.70-35.64) | No | 5.00 (3.19) | No | 4.99 (0.97) | No | 2.32 (0.65) | Yes |
| Hypersexuality | 3 | 4.99 (1.61-15.51) | Yes | 4.99 (9.55) | Yes | 4.98 (1.93) | No | 2.32 (0.65) | Yes |
| Illusion | 3 | 4.98 (1.60-15.46) | Yes | 4.98 (9.50) | Yes | 4.96 (1.92) | No | 2.31 (0.64) | Yes |
| Grief reaction | 1 | 4.89 (0.69-34.85) | No | 4.89 (3.09) | No | 4.88 (0.94) | No | 2.29 (0.61) | Yes |
| Intentional overdose | 119 | 4.83 (4.03-5.78) | Yes | 4.81 (358.02) | Yes | 4.80 (4.12) | Yes | 2.26 (0.60) | Yes |
| Sedation complication | 6 | 4.81 (2.16-10.73) | Yes | 4.81 (18.07) | Yes | 4.80 (2.46) | Yes | 2.26 (0.60) | Yes |
| Patient dissatisfaction with treatment | 3 | 4.78 (1.54-14.86) | Yes | 4.78 (8.95) | Yes | 4.77 (1.85) | No | 2.25 (0.59) | Yes |
| Dissociation | 9 | 4.77 (2.48-9.17) | Yes | 4.76 (26.69) | Yes | 4.75 (2.75) | Yes | 2.25 (0.58) | Yes |
| No adverse event | 315 | 4.70 (4.21-5.26) | Yes | 4.66 (903.97) | Yes | 4.64 (4.23) | Yes | 2.22 (0.55) | Yes |
| Food interaction | 3 | 4.70 (1.51-14.59) | Yes | 4.70 (8.70) | Yes | 4.69 (1.82) | No | 2.23 (0.56) | Yes |
| Facial spasm | 2 | 4.70 (1.17-18.84) | No | 4.70 (5.81) | No | 4.69 (1.47) | No | 2.23 (0.56) | Yes |
| Indifference | 1 | 4.63 (0.65-32.98) | No | 4.63 (2.84) | No | 4.62 (0.89) | No | 2.21 (0.53) | Yes |
| Drug screen positive | 10 | 4.62 (2.48-8.59) | Yes | 4.62 (28.24) | Yes | 4.60 (2.74) | Yes | 2.20 (0.54) | Yes |
| Foaming at mouth | 3 | 4.58 (1.48-14.23) | Yes | 4.58 (8.38) | Yes | 4.57 (1.77) | No | 2.19 (0.52) | Yes |
| Libido increased | 3 | 4.56 (1.47-14.16) | Yes | 4.56 (8.31) | Yes | 4.55 (1.76) | No | 2.19 (0.52) | Yes |
| Concussion | 14 | 4.54 (2.69-7.67) | Yes | 4.54 (38.51) | Yes | 4.53 (2.92) | Yes | 2.18 (0.51) | Yes |
| Movement disorder | 50 | 4.51 (3.42-5.96) | Yes | 4.51 (136.09) | Yes | 4.50 (3.56) | Yes | 2.17 (0.50) | Yes |
| Facial discomfort | 1 | 4.51 (0.63-32.12) | No | 4.51 (2.72) | No | 4.50 (0.87) | No | 2.17 (0.50) | Yes |
| Eye contusion | 5 | 4.50 (1.87-10.82) | Yes | 4.50 (13.55) | Yes | 4.49 (2.15) | Yes | 2.17 (0.50) | Yes |
| Orthopaedic procedure | 1 | 4.49 (0.63-31.93) | No | 4.48 (2.70) | No | 4.47 (0.87) | No | 2.16 (0.49) | Yes |
| Circumstance or information capable of leading to medication error | 61 | 4.47 (3.47-5.75) | Yes | 4.46 (163.34) | Yes | 4.45 (3.60) | Yes | 2.15 (0.49) | Yes |
| Pressure of speech | 1 | 4.46 (0.63-31.75) | No | 4.46 (2.68) | No | 4.45 (0.86) | No | 2.15 (0.48) | Yes |
| Degenerative bone disease | 1 | 4.45 (0.62-31.66) | No | 4.45 (2.66) | No | 4.44 (0.86) | No | 2.15 (0.48) | Yes |
| Depressive symptom | 6 | 4.44 (1.99-9.90) | Yes | 4.44 (15.95) | Yes | 4.43 (2.27) | Yes | 2.15 (0.48) | Yes |
| Mental fatigue | 3 | 4.39 (1.41-13.64) | Yes | 4.39 (7.84) | Yes | 4.38 (1.70) | No | 2.13 (0.46) | Yes |
| Sedation | 39 | 4.38 (3.20-5.99) | Yes | 4.37 (101.13) | Yes | 4.36 (3.35) | Yes | 2.12 (0.46) | Yes |
| Persistent depressive disorder | 1 | 4.31 (0.61-30.69) | No | 4.31 (2.54) | No | 4.30 (0.83) | No | 2.10 (0.43) | Yes |
| Violence-related symptom | 2 | 4.30 (1.07-17.23) | No | 4.30 (5.05) | No | 4.29 (1.34) | No | 2.10 (0.43) | Yes |
| Night sweats | 52 | 4.25 (3.24-5.59) | Yes | 4.25 (128.85) | Yes | 4.24 (3.37) | Yes | 2.08 (0.42) | Yes |
| Change of bowel habit | 3 | 4.22 (1.36-13.11) | Yes | 4.22 (7.36) | Yes | 4.21 (1.63) | No | 2.07 (0.41) | Yes |
| Conversion disorder | 2 | 4.22 (1.05-16.90) | No | 4.22 (4.90) | No | 4.21 (1.32) | No | 2.07 (0.40) | Yes |
| Physical product label issue | 2 | 4.18 (1.04-16.76) | No | 4.18 (4.83) | No | 4.18 (1.31) | No | 2.06 (0.39) | Yes |
| Diet refusal | 1 | 4.18 (0.59-29.78) | No | 4.18 (2.42) | No | 4.18 (0.81) | No | 2.06 (0.39) | Yes |
| Head titubation | 2 | 4.16 (1.04-16.65) | No | 4.16 (4.78) | No | 4.15 (1.30) | No | 2.05 (0.38) | Yes |
| Product prescribing issue | 27 | 4.15 (2.84-6.05) | Yes | 4.14 (64.22) | Yes | 4.13 (3.01) | Yes | 2.05 (0.38) | Yes |
| Nail injury | 1 | 4.12 (0.58-29.31) | No | 4.12 (2.35) | No | 4.11 (0.80) | No | 2.04 (0.37) | Yes |
| Manufacturing issue | 1 | 4.12 (0.58-29.31) | No | 4.12 (2.35) | No | 4.11 (0.80) | No | 2.04 (0.37) | Yes |
| Vulval cancer | 1 | 4.11 (0.58-29.23) | No | 4.11 (2.34) | No | 4.10 (0.79) | No | 2.03 (0.36) | Yes |
| Alcohol use | 3 | 4.05 (1.30-12.58) | Yes | 4.05 (6.87) | Yes | 4.04 (1.57) | No | 2.01 (0.35) | Yes |
| Trichotillomania | 1 | 4.05 (0.57-28.85) | No | 4.05 (2.29) | No | 4.04 (0.78) | No | 2.02 (0.34) | Yes |
| Counterfeit product administered | 2 | 4.02 (1.00-16.11) | No | 4.02 (4.53) | No | 4.01 (1.26) | No | 2.00 (0.34) | Yes |
| Hypermagnesaemia | 1 | 4.02 (0.57-28.62) | No | 4.02 (2.26) | No | 4.01 (0.78) | No | 2.00 (0.33) | Yes |
| Lip exfoliation | 2 | 4.00 (1.00-16.01) | No | 4.00 (4.48) | No | 3.99 (1.25) | No | 2.00 (0.33) | Yes |
| Head discomfort | 32 | 3.97 (2.80-5.61) | Yes | 3.96 (70.76) | Yes | 3.96 (2.96) | Yes | 1.98 (0.32) | Yes |
| Drug effect less than expected | 16 | 3.97 (2.43-6.48) | Yes | 3.97 (35.40) | Yes | 3.96 (2.63) | Yes | 1.98 (0.32) | Yes |
| Abnormal behaviour | 49 | 3.94 (2.98-5.22) | Yes | 3.93 (106.99) | Yes | 3.93 (3.10) | Yes | 1.97 (0.31) | Yes |
| Drug tolerance | 8 | 3.90 (1.95-7.80) | Yes | 3.89 (17.17) | Yes | 3.89 (2.17) | Yes | 1.96 (0.29) | Yes |
| Helicobacter infection | 14 | 3.88 (2.30-6.56) | Yes | 3.88 (29.83) | Yes | 3.87 (2.50) | Yes | 1.95 (0.29) | Yes |
| Altered visual depth perception | 1 | 3.88 (0.55-27.62) | No | 3.88 (2.13) | No | 3.87 (0.75) | No | 1.95 (0.28) | Yes |
| Alcohol poisoning | 3 | 3.86 (1.24-11.97) | Yes | 3.86 (6.33) | Yes | 3.85 (1.49) | No | 1.94 (0.28) | Yes |
| Lipids abnormal | 1 | 3.86 (0.54-27.48) | No | 3.86 (2.12) | No | 3.85 (0.75) | No | 1.95 (0.27) | Yes |
| Peptic ulcer haemorrhage | 1 | 3.86 (0.54-27.48) | No | 3.86 (2.12) | No | 3.85 (0.75) | No | 1.95 (0.27) | Yes |
| Hallucination, visual | 28 | 3.85 (2.66-5.58) | Yes | 3.85 (58.87) | Yes | 3.84 (2.82) | Yes | 1.94 (0.27) | Yes |
| Product tampering | 2 | 3.82 (0.95-15.30) | No | 3.82 (4.15) | No | 3.81 (1.19) | No | 1.93 (0.26) | Yes |
| Dry mouth | 113 | 3.81 (3.16-4.58) | Yes | 3.79 (232.15) | Yes | 3.79 (3.24) | Yes | 1.92 (0.25) | Yes |
| Product design issue | 1 | 3.81 (0.54-27.15) | No | 3.81 (2.07) | No | 3.81 (0.74) | No | 1.93 (0.26) | Yes |
| Poor quality product administered | 25 | 3.80 (2.57-5.63) | Yes | 3.8 (51.39) | Yes | 3.79 (2.73) | Yes | 1.92 (0.26) | Yes |
| Head and neck cancer | 1 | 3.80 (0.53-27.01) | No | 3.80 (2.05) | No | 3.79 (0.73) | No | 1.92 (0.25) | Yes |
| Bladder irritation | 1 | 3.79 (0.53-26.95) | No | 3.79 (2.05) | No | 3.78 (0.73) | No | 1.92 (0.25) | Yes |
| Extensor plantar response | 1 | 3.78 (0.53-26.88) | No | 3.78 (2.04) | No | 3.77 (0.73) | No | 1.91 (0.24) | Yes |
| Judgement impaired | 2 | 3.76 (0.94-15.08) | No | 3.76 (4.05) | No | 3.76 (1.18) | No | 1.91 (0.24) | Yes |
| Joint ankylosis | 1 | 3.72 (0.52-26.50) | No | 3.72 (1.99) | No | 3.72 (0.72) | No | 1.89 (0.22) | Yes |
| Product supply issue | 12 | 3.71 (2.10-6.54) | Yes | 3.71 (23.68) | Yes | 3.70 (2.30) | Yes | 1.89 (0.22) | Yes |
| Yawning | 4 | 3.71 (1.39-9.89) | Yes | 3.71 (7.88) | Yes | 3.70 (1.63) | No | 1.89 (0.22) | Yes |
| Gallbladder polyp | 1 | 3.71 (0.52-26.43) | No | 3.71 (1.98) | No | 3.71 (0.72) | No | 1.89 (0.22) | Yes |
| Renal aplasia | 1 | 3.71 (0.52-26.37) | No | 3.71 (1.97) | No | 3.70 (0.72) | No | 1.89 (0.22) | Yes |
| Product colour issue | 8 | 3.67 (1.84-7.35) | Yes | 3.67 (15.53) | Yes | 3.67 (2.05) | Yes | 1.87 (0.21) | Yes |
| Palpitations | 158 | 3.64 (3.11-4.26) | Yes | 3.62 (299.72) | Yes | 3.62 (3.17) | Yes | 1.85 (0.19) | Yes |
| Transient global amnesia | 1 | 3.64 (0.51-25.88) | No | 3.64 (1.91) | No | 3.63 (0.70) | No | 1.86 (0.19) | Yes |
| Alcohol withdrawal syndrome | 1 | 3.64 (0.51-25.94) | No | 3.64 (1.91) | No | 3.64 (0.70) | No | 1.86 (0.19) | Yes |
| Bronchitis viral | 1 | 3.63 (0.51-25.81) | No | 3.63 (1.90) | No | 3.62 (0.70) | No | 1.86 (0.18) | Yes |
| Migraine with aura | 4 | 3.61 (1.35-9.64) | Yes | 3.61 (7.54) | Yes | 3.61 (1.59) | No | 1.85 (0.18) | Yes |
| Hyperventilation | 7 | 3.57 (1.70-7.50) | Yes | 3.57 (12.93) | Yes | 3.56 (1.92) | No | 1.83 (0.17) | Yes |
| Central obesity | 1 | 3.54 (0.50-25.17) | No | 3.54 (1.82) | No | 3.53 (0.68) | No | 1.82 (0.15) | Yes |
| Panic disorder | 5 | 3.53 (1.47-8.50) | Yes | 3.53 (9.06) | Yes | 3.53 (1.69) | No | 1.82 (0.15) | Yes |
| Brain contusion | 1 | 3.53 (0.50-25.11) | No | 3.53 (1.81) | No | 3.52 (0.68) | No | 1.82 (0.15) | Yes |
| Suicidal ideation | 109 | 3.49 (2.89-4.21) | Yes | 3.48 (192.26) | Yes | 3.47 (2.97) | Yes | 1.80 (0.13) | Yes |
| Photopsia | 8 | 3.46 (1.73-6.92) | Yes | 3.46 (13.94) | Yes | 3.45 (1.93) | No | 1.79 (0.12) | Yes |
| Joint instability | 2 | 3.46 (0.86-13.85) | No | 3.46 (3.49) | No | 3.45 (1.08) | No | 1.79 (0.12) | Yes |
| Drug-disease interaction | 1 | 3.46 (0.49-24.61) | No | 3.46 (1.74) | No | 3.45 (0.67) | No | 1.79 (0.12) | Yes |
| Product dispensing issue | 3 | 3.44 (1.11-10.68) | Yes | 3.44 (5.18) | Yes | 3.44 (1.33) | No | 1.78 (0.11) | Yes |
| Euphoric mood | 13 | 3.42 (1.98-5.89) | Yes | 3.42 (22.17) | Yes | 3.41 (2.16) | Yes | 1.77 (0.10) | Yes |
| Nervousness | 65 | 3.40 (2.66-4.34) | Yes | 3.39 (109.55) | Yes | 3.39 (2.76) | Yes | 1.76 (0.09) | Yes |
| Amnesia | 76 | 3.30 (2.64-4.14) | Yes | 3.30 (121.53) | Yes | 3.29 (2.73) | Yes | 1.72 (0.05) | Yes |
| Product packaging issue | 14 | 3.28 (1.94-5.54) | Yes | 3.28 (22.15) | Yes | 3.28 (2.11) | Yes | 1.71 (0.05) | Yes |
| Acute promyelocytic leukaemia | 1 | 3.28 (0.46-23.31) | No | 3.28 (1.58) | No | 3.27 (0.63) | No | 1.71 (0.04) | Yes |
| Prescription drug used without a prescription | 9 | 3.27 (1.70-6.29) | Yes | 3.27 (14.15) | Yes | 3.26 (1.89) | No | 1.71 (0.04) | Yes |
| Cardiac infection | 1 | 3.23 (0.45-22.97) | No | 3.23 (1.54) | No | 3.22 (0.62) | No | 1.69 (0.02) | Yes |
| Prescribed overdose | 27 | 3.22 (2.20-4.69) | Yes | 3.21 (41.11) | Yes | 3.21 (2.34) | Yes | 1.68 (0.02) | Yes |
| Cortisol increased | 1 | 3.22 (0.45-22.92) | No | 3.22 (1.53) | No | 3.22 (0.62) | No | 1.69 (0.01) | Yes |
| Monoplegia | 5 | 3.21 (1.34-7.73) | Yes | 3.21 (7.61) | Yes | 3.21 (1.54) | No | 1.68 (0.02) | Yes |
| Family stress | 1 | 3.20 (0.45-22.73) | No | 3.20 (1.51) | No | 3.19 (0.62) | No | 1.67 (0.00) | Yes |
| Vertigo positional | 2 | 3.19 (0.80-12.76) | No | 3.19 (2.99) | No | 3.18 (1.00) | No | 1.67 (0.00) | Yes |
| Incorrect dosage administered | 8 | 3.18 (1.59-6.36) | Yes | 3.18 (11.92) | Yes | 3.17 (1.78) | No | 1.67 (0.00) | Yes |
| Wrong technique in product usage process | 341 | 3.16 (2.84-3.51) | Yes | 3.13 (494.33) | Yes | 3.12 (2.85) | Yes | 1.64 (-0.02) | No |
| Disorientation | 42 | 3.16 (2.33-4.27) | Yes | 3.15 (61.68) | Yes | 3.15 (2.44) | Yes | 1.66 (-0.01) | No |
| Anxiety disorder | 4 | 3.08 (1.16-8.22) | Yes | 3.08 (5.62) | Yes | 3.08 (1.35) | No | 1.62 (-0.04) | No |
| Bipolar disorder | 11 | 3.06 (1.69-5.53) | Yes | 3.06 (15.25) | Yes | 3.06 (1.86) | No | 1.61 (-0.05) | No |
| Depressed level of consciousness | 41 | 3.02 (2.22-4.11) | Yes | 3.02 (55.26) | Yes | 3.01 (2.33) | Yes | 1.59 (-0.07) | No |
| Panic attack | 39 | 3.02 (2.21-4.14) | Yes | 3.02 (52.61) | Yes | 3.02 (2.32) | Yes | 1.59 (-0.07) | No |
| Self-injurious ideation | 4 | 3.01 (1.13-8.03) | Yes | 3.01 (5.35) | Yes | 3.00 (1.32) | No | 1.59 (-0.08) | No |
| Craniofacial fracture | 5 | 2.98 (1.24-7.16) | Yes | 2.98 (6.55) | Yes | 2.97 (1.43) | No | 1.57 (-0.09) | No |
| Hyperphagia | 4 | 2.95 (1.11-7.87) | Yes | 2.95 (5.15) | Yes | 2.95 (1.30) | No | 1.56 (-0.11) | No |
| Myoclonus | 12 | 2.92 (1.66-5.14) | Yes | 2.92 (15.09) | Yes | 2.91 (1.81) | No | 1.54 (-0.12) | No |
| Micturition disorder | 4 | 2.90 (1.09-7.74) | Yes | 2.90 (4.98) | Yes | 2.90 (1.28) | No | 1.54 (-0.13) | No |
| Therapeutic product effect decreased | 112 | 2.72 (2.25-3.27) | Yes | 2.71 (120.57) | Yes | 2.70 (2.31) | Yes | 1.44 (-0.23) | No |
| Suicide attempt | 55 | 2.66 (2.04-3.46) | Yes | 2.65 (56.66) | Yes | 2.65 (2.12) | Yes | 1.41 (-0.26) | No |
| Crying | 33 | 2.58 (1.83-3.63) | Yes | 2.58 (31.77) | Yes | 2.57 (1.93) | No | 1.36 (-0.30) | No |
| Anger | 31 | 2.55 (1.80-3.63) | Yes | 2.55 (29.25) | Yes | 2.55 (1.90) | No | 1.35 (-0.32) | No |
| Mental impairment | 23 | 2.54 (1.69-3.82) | Yes | 2.54 (21.41) | Yes | 2.54 (1.80) | No | 1.34 (-0.32) | No |
| Frustration tolerance decreased | 13 | 2.53 (1.47-4.35) | Yes | 2.53 (11.97) | Yes | 2.52 (1.60) | No | 1.34 (-0.33) | No |
| Paranoia | 14 | 2.51 (1.48-4.23) | Yes | 2.50 (12.64) | Yes | 2.50 (1.61) | No | 1.32 (-0.34) | No |
| Irritability | 55 | 2.46 (1.89-3.20) | Yes | 2.46 (47.41) | Yes | 2.45 (1.97) | No | 1.29 (-0.37) | No |
| Anxiety | 268 | 2.45 (2.17-2.76) | Yes | 2.43 (226.21) | Yes | 2.43 (2.20) | Yes | 1.28 (-0.39) | No |
| Respiratory depression | 11 | 2.45 (1.36-4.43) | Yes | 2.45 (9.43) | Yes | 2.45 (1.49) | No | 1.29 (-0.37) | No |
| Headache | 606 | 2.43 (2.24-2.64) | Yes | 2.40 (497.39) | Yes | 2.39 (2.24) | Yes | 1.26 (-0.41) | No |
| Pollakiuria | 39 | 2.42 (1.77-3.32) | Yes | 2.42 (32.45) | Yes | 2.42 (1.86) | No | 1.27 (-0.39) | No |
| Disturbance in attention | 49 | 2.36 (1.78-3.13) | Yes | 2.36 (38.33) | Yes | 2.36 (1.86) | No | 1.24 (-0.43) | No |
| Apnoea | 6 | 2.36 (1.06-5.27) | Yes | 2.36 (4.72) | Yes | 2.36 (1.21) | No | 1.24 (-0.43) | No |
| Inappropriate schedule of product administration | 264 | 2.35 (2.08-2.65) | Yes | 2.34 (202.5) | Yes | 2.33 (2.11) | Yes | 1.22 (-0.44) | No |
| Lethargy | 49 | 2.33 (1.76-3.08) | Yes | 2.32 (36.92) | Yes | 2.32 (1.84) | No | 1.22 (-0.45) | No |
| Therapeutic response unexpected | 47 | 2.23 (1.68-2.97) | Yes | 2.23 (31.87) | Yes | 2.23 (1.75) | No | 1.16 (-0.51) | No |
| Cold sweat | 14 | 2.23 (1.32-3.77) | Yes | 2.23 (9.51) | Yes | 2.23 (1.44) | No | 1.16 (-0.51) | No |
| Hypoaesthesia oral | 12 | 2.22 (1.26-3.91) | Yes | 2.22 (8.00) | Yes | 2.21 (1.38) | No | 1.15 (-0.52) | No |
| Mood altered | 22 | 2.19 (1.44-3.33) | Yes | 2.19 (14.27) | Yes | 2.19 (1.54) | No | 1.13 (-0.53) | No |
| Urinary retention | 27 | 2.18 (1.49-3.17) | Yes | 2.17 (17.12) | Yes | 2.17 (1.58) | No | 1.12 (-0.55) | No |
| Tinnitus | 38 | 2.17 (1.58-2.99) | Yes | 2.17 (24.03) | Yes | 2.17 (1.66) | No | 1.12 (-0.55) | No |
| Pharyngeal oedema | 11 | 2.16 (1.20-3.91) | Yes | 2.16 (6.88) | Yes | 2.16 (1.32) | No | 1.11 (-0.55) | No |
| Muscle twitching | 18 | 2.14 (1.35-3.40) | Yes | 2.14 (10.93) | Yes | 2.14 (1.45) | No | 1.10 (-0.57) | No |
| Adverse reaction | 13 | 2.14 (1.24-3.68) | Yes | 2.14 (7.84) | Yes | 2.13 (1.35) | No | 1.09 (-0.57) | No |
| Scratch | 8 | 2.07 (1.04-4.15) | Yes | 2.07 (4.43) | Yes | 2.07 (1.16) | No | 1.05 (-0.62) | No |
| Depression | 168 | 2.06 (1.77-2.40) | Yes | 2.05 (91.10) | Yes | 2.05 (1.81) | No | 1.04 (-0.63) | No |
| Neuroleptic malignant syndrome | 8 | 2.02 (1.01-4.03) | Yes | 2.02 (4.09) | Yes | 2.01 (1.13) | No | 1.01 (-0.66) | No |
| Head injury | 24 | 2.01 (1.34-2.99) | Yes | 2.00 (12.08) | Yes | 2.00 (1.43) | No | 1.00 (-0.66) | No |
| Aggression | 32 | 2.00 (1.41-2.83) | Yes | 2.00 (15.90) | Yes | 2.00 (1.49) | No | 1.00 (-0.67) | No |
| Thirst | 14 | 1.99 (1.18-3.36) | Yes | 1.99 (6.90) | No | 1.99 (1.28) | No | 0.99 (-0.67) | No |
| Tremor | 118 | 1.94 (1.62-2.32) | Yes | 1.94 (53.41) | No | 1.93 (1.66) | No | 0.95 (-0.71) | No |
| Dyskinesia | 27 | 1.92 (1.32-2.80) | Yes | 1.92 (11.89) | No | 1.92 (1.40) | No | 0.94 (-0.73) | No |
| Depressed mood | 38 | 1.78 (1.29-2.45) | Yes | 1.78 (12.92) | No | 1.78 (1.36) | No | 0.83 (-0.84) | No |
| Aphasia | 20 | 1.78 (1.15-2.76) | Yes | 1.78 (6.85) | No | 1.78 (1.23) | No | 0.83 (-0.83) | No |
| Illness | 67 | 1.75 (1.38-2.23) | Yes | 1.75 (21.61) | No | 1.75 (1.43) | No | 0.81 (-0.86) | No |
| Confusional state | 103 | 1.72 (1.42-2.09) | Yes | 1.72 (30.8) | No | 1.72 (1.46) | No | 0.78 (-0.89) | No |
| Accidental overdose | 22 | 1.69 (1.11-2.56) | Yes | 1.69 (6.14) | No | 1.69 (1.19) | No | 0.75 (-0.91) | No |
| Muscular weakness | 70 | 1.65 (1.31-2.09) | Yes | 1.65 (18.05) | No | 1.65 (1.36) | No | 0.72 (-0.94) | No |
| Migraine | 63 | 1.65 (1.29-2.11) | Yes | 1.65 (16.02) | No | 1.65 (1.34) | No | 0.72 (-0.95) | No |
| Road traffic accident | 24 | 1.64 (1.10-2.45) | Yes | 1.64 (6.05) | No | 1.64 (1.18) | No | 0.72 (-0.95) | No |
| Psychotic disorder | 16 | 1.64 (1.01-2.69) | Yes | 1.64 (4.04) | No | 1.64 (1.09) | No | 0.72 (-0.95) | No |
| Mental disorder | 26 | 1.62 (1.10-2.38) | Yes | 1.62 (6.11) | No | 1.62 (1.17) | No | 0.69 (-0.97) | No |
| Memory impairment | 92 | 1.58 (1.29-1.94) | Yes | 1.58 (19.74) | No | 1.58 (1.33) | No | 0.66 (-1.00) | No |
| Dizziness | 295 | 1.55 (1.38-1.73) | Yes | 1.54 (56.16) | No | 1.54 (1.40) | No | 0.62 (-1.04) | No |
| Loss of personal independence in daily activities | 44 | 1.53 (1.14-2.06) | Yes | 1.53 (8.15) | No | 1.53 (1.20) | No | 0.62 (-1.05) | No |
| Heart rate increased | 55 | 1.50 (1.15-1.96) | Yes | 1.50 (9.16) | No | 1.50 (1.20) | No | 0.58 (-1.08) | No |
| Speech disorder | 29 | 1.50 (1.04-2.16) | Yes | 1.50 (4.87) | No | 1.50 (1.11) | No | 0.59 (-1.08) | No |
| Overdose | 147 | 1.48 (1.26-1.74) | Yes | 1.48 (22.66) | No | 1.48 (1.29) | No | 0.56 (-1.10) | No |
| Therapeutic response decreased | 32 | 1.44 (1.01-2.03) | Yes | 1.43 (4.21) | No | 1.43 (1.07) | No | 0.52 (-1.15) | No |
| Hyperhidrosis | 69 | 1.42 (1.12-1.80) | Yes | 1.42 (8.58) | No | 1.42 (1.16) | No | 0.51 (-1.16) | No |
| Product dose omission issue | 320 | 1.39 (1.24-1.55) | Yes | 1.38 (34.09) | No | 1.38 (1.26) | No | 0.47 (-1.20) | No |
| Underdose | 49 | 1.37 (1.04-1.82) | Yes | 1.37 (4.94) | No | 1.37 (1.08) | No | 0.46 (-1.21) | No |
| Product use issue | 130 | 1.35 (1.13-1.60) | Yes | 1.34 (11.46) | No | 1.34 (1.16) | No | 0.43 (-1.24) | No |
| Product quality issue | 69 | 1.28 (1.01-1.62) | Yes | 1.28 (4.28) | No | 1.28 (1.05) | No | 0.36 (-1.31) | No |
| Fatigue | 379 | 1.16 (1.05-1.29) | Yes | 1.16 (8.66) | No | 1.16 (1.07) | No | 0.22 (-1.45) | No |
